# Allosteric Control Overcomes Steric Limitations for Neutralizing Antibodies Targeting Conserved Binding Epitopes of the SARS-CoV-2 Spike Protein: Exploring the Intersection of Binding, Allostery, and Immune Escape with a Multimodal Computational Approach

**DOI:** 10.3390/biom15091340

**Published:** 2025-09-18

**Authors:** Mohammed Alshahrani, Vedant Parikh, Brandon Foley, Gennady Verkhivker

**Affiliations:** 1 Keck Center for Science and Engineering, Graduate Program in Computational and Data Sciences, Schmid College of Science and Technology, Chapman University, Orange, CA 92866, USA; alshahrani@chapman.edu (M.A.); vedpar31@gmail.com (V.P.); brfoley@chapman.edu (B.F.); 2 Department of Biomedical and Pharmaceutical Sciences, Chapman University School of Pharmacy, Irvine, CA 92618, USA

**Keywords:** SARS-CoV-2 spike protein, Omicron variants, antibody binding, immune escape, molecular dynamics, protein stability, mutational scanning, binding energetics, evolutionary mechanisms

## Abstract

Understanding the atomistic basis of multi-layer mechanisms employed by broadly reactive neutralizing antibodies of the SARS-CoV-2 spike protein without directly blocking receptor engagement remains an important challenge in coronavirus immunology. Class 4 antibodies represent an intriguing case: they target a deeply conserved, cryptic epitope on the receptor-binding domain yet exhibit variable neutralization potency across subgroups F1 (CR3022, EY6A, COVA1-16), F2 (DH1047), and F3 (S2X259). The molecular basis for this variability is not fully understood. Here, we employed a multi-modal computational approach integrating atomistic and coarse-grained molecular dynamics simulations, binding free energy calculations, mutational scanning, and dynamic network analysis to elucidate how these antibodies engage the receptor-binding domain (RBD) of the SARS-CoV-2 spike protein and influence its function. Our results reveal that neutralization efficacy arises from the interplay of direct interfacial interactions and allosteric effects. Group F1 antibodies (CR3022, EY6A, COVA1-16) primarily operate via classic allostery, modulating flexibility in RBD loop regions to indirectly interfere with the ACE2 receptor binding through long-range effects. Group F2 antibody DH1047 represents an intermediate mechanism, combining partial steric hindrance—through engagement of ACE2-critical residues T376, R408, V503, and Y508—with significant allosteric influence, facilitated by localized communication pathways linking the epitope to the receptor interface. Group F3 antibody S2X259 achieves potent neutralization through a synergistic mechanism involving direct competition with ACE2 and localized allosteric stabilization, albeit with potentially increased escape vulnerability. Dynamic network analysis identified a conserved “allosteric ring” within the RBD core that serves as a structural scaffold for long-range signal propagation, with antibody-specific extensions modulating communication to the ACE2 interface. These findings support a model where Class 4 neutralization strategies evolve through the refinement of peripheral allosteric connections rather than epitope redesign. This study establishes a robust computational framework for understanding the atomistic basis of neutralization activity and immune escape for Class 4 antibodies, highlighting how the interplay of binding energetics, conformational dynamics, and allosteric modulation governs their effectiveness against SARS-CoV-2.

## 1. Introduction

The emergence of severe acute respiratory syndrome coronavirus 2 (SARS-CoV-2) has triggered an unprecedented surge in global research efforts aimed at deciphering its molecular architecture, mechanisms of cellular invasion, and the immune responses it elicits. Central to these investigations is the SARS-CoV-2 Spike (S) glycoprotein, a trimeric surface protein that plays a pivotal role in mediating viral entry into host cells and serves as the primary target for neutralizing antibodies [1,2,3,4,5,6,7,8,9,10,11,12,13,14,15]. The S protein exhibits extraordinary conformational plasticity, enabling it to navigate through multiple functional states—from receptor engagement to membrane fusion—while simultaneously evading immune surveillance. Structurally, the S protein is composed of two functionally distinct subunits: S1 and S2. The S1 subunit contains four key domains—the N-terminal domain (NTD), the RBD, and two conserved subdomains, SD1 and SD2—each contributing uniquely to the dynamic life cycle of the virus. The NTD is implicated in early stages of host cell recognition, potentially facilitating attachment via interactions with glycans or other cell surface components [1,2,3,4,5,6,7,8,9,10,11,12,13,14,15]. The RBD binds to the angiotensin-converting enzyme 2 (ACE2) receptor, transitioning between “up” and “down” conformations to modulate receptor and antibody accessibility [1,2,3,4,5,6,7,8,9,10,11,12,13,14,15]. The SD1 and SD2 subdomains play essential structural roles in maintaining the prefusion conformation of the S protein, acting as molecular scaffolds that regulate the timing and efficiency of membrane fusion [10,11,12,13,14,15,16,17,18]. Detailed biophysical analyses have illuminated the thermodynamic and kinetic underpinnings of these conformational transitions, revealing how subtle energetic shifts govern large-scale structural rearrangements critical for viral infectivity [16,17,18]. The rapid evolution of SARS-CoV-2 variants of concern (VOCs) has underscored the importance of understanding how mutations in the S protein affect antigenicity and transmissibility. Cryo-electron microscopy (cryo-EM) and X-ray crystallography studies generated an extensive structural atlas of the S protein in various functional states, including complexes formed with neutralizing antibodies [19,20,21,22,23,24,25].

A cornerstone of the adaptive immune response to SARS-CoV-2 is the production of neutralizing antibodies targeting the S protein, especially those directed against the RBD. High-throughput yeast display screening and deep mutational scanning (DMS) have revolutionized our ability to dissect the antigenic landscape of the RBD at near-residue resolution [26]. Using 247 broadly neutralizing antibodies against sarbecoviruses, recent studies employed high-throughput yeast-DMS experiments to determine the RBD escape mutation profiles. Based on the epitope specificity, these antibodies were subsequently classified into six major groups (A–F) [26]. This classification was further expanded using data from Omicron BA.1 variant infections in vaccinated individuals, identifying 12 distinct epitope groups among 1640 RBD-targeting antibodies [27]. This refined categorization offered a more detailed understanding of how different antibody classes interact with the RBD and adapt to new mutations. Among these groups, groups A–C are characterized by antibodies that bind directly within the ACE2-binding RBD regions, making them highly effective at blocking viral attachment. However, they are also the most vulnerable to escape mutations, particularly at residues K417, E484, and N501, which are frequently altered in VOCs [27]. Group D antibodies, exemplified by monoclonals such as REGN-10987, LY-CoV1404, and COV2-2130, recognize a distinct epitope centered around residues 440–449 on the RBD.

Groups E and F represent antibodies that recognize epitopes outside the central ACE2-binding site, offering broader protection due to their less direct overlap with the receptor interface. These groups were further subdivided into E1–E3 and F1–F3, respectively, covering both the front and back surfaces of the RBD, thereby providing a comprehensive view of non-competitive epitopes. In this classification, group E1 antibodies target the S309 epitope [28,29], group E3 antibodies target the S2H97 binding site [30], group F1 antibodies recognize the CR3022/S304 epitope [31,32], group F2 antibodies bind to the DH1047 site [33], and group F3 antibodies bind to the ADG-2 site [34,35]. The epitope clusters E and F correspond to Class 3 and Class 4 antibodies in earlier taxonomies [36]. The latest seminal studies analyzed the escape mutation profiles of a total of 2688 monoclonal antibodies including 1874 antibodies isolated from individuals infected with XBB or JN.1 variants, ultimately resulting in the identification of 22 distinct antibody clusters and a detailed antigenic map of the RBD [37,38]. Importantly, this pioneering study showed the possibility of accurately predicting SARS-CoV-2 RBD evolution by aggregating high-throughput antibody DMS results and constructing pseudoviruses that carry the predicted mutations as filters to screen for potent neutralizing antibodies. Discoveries of E1 and F3 antibodies revealed that group F3 antibody SA55 and group E1 antibody SA58 can bind non-competitively to the RBD with robust neutralizing activity against a broad range of immune-evading variants [39,40]. Another large-scale investigation employing high-throughput DMS assays for 1637 potent monoclonal antibodies evaluated immune escape across eight major SARS-CoV-2 variants, including B.1 (D614G), Omicron BA.1, BA.2, BA.5, BQ.1.1, XBB.1.5, HK.3, and JN.1 [41]. Pan-sarbecovirus binding assays combined with in vitro mapping of viral escape, structural analyses, and DMS experiments provided a comprehensive characterization of a panel of antibodies targeting different epitopes, including a conserved cryptic RBD region [42]. In a recent breakthrough investigation, the two antibodies CYFN1006-1 and CYFN1006-2 demonstrated superior neutralization breadth across all tested SARS-CoV-2 variants, even outperforming SA55 [43]. These investigations have systematically cataloged RBD escape mutations associated with a wide array of monoclonal antibodies, revealing distinct patterns of escape-prone regions and mutation-resistant epitopes.

Computational approaches have played a transformative role in elucidating the structural dynamics and functional mechanisms of the SARS-CoV-2 S protein at atomic resolution. These tools have enabled researchers to probe not only the intrinsic flexibility of the S protein but also its interactions with key molecular partners such as the ACE2 receptor and a wide array of neutralizing antibodies. Through advanced simulation techniques—including molecular dynamics (MD) simulations and Markov state models (MSMs)—the conformational landscapes of Omicron subvariants such as XBB.1 and XBB.1.5, both in their free forms and bound to ACE2 or antibodies, have been systematically characterized [44]. These studies have provided critical insights into how mutations modulate conformational transitions, stability, and receptor accessibility across evolving variants. In parallel, computational mutational scanning and binding affinity analyses have offered a quantitative framework for interpreting experimental observations related to the interaction between Omicron XBB variants and ACE2, as well as their susceptibility to a panel of Class 1 neutralizing antibodies [45,46]. These approaches have proven instrumental in dissecting the molecular basis of immune escape and viral adaptation. Building on these advances, our group has integrated AlphaFold2-based atomistic modeling with ensemble sampling methods to predict and analyze the structures and conformational ensembles of S-ACE2 complexes across the most prevalent Omicron subvariants, including JN.1, KP.1, KP.2, and KP.3 [47]. Recent investigations uncovered a multifactor-based mechanism that governs the emergence of escape mutants against ultra-potent monoclonal antibodies [48,49]. In the proposed mechanism, the selection of specific mutations is driven by an intricate interplay between their effects on protein structural stability, binding affinity, and long-range allosteric communication networks within the RBD. Further computational efforts have focused on understanding the mechanisms of broadly neutralizing antibodies, particularly those belonging to the E1 and F3 groups [50]. A recent comparative modeling study shed light on the distinct molecular strategies employed by several broadly neutralizing antibodies, including S309, S304, CYFN1006, and VIR-7229 [51]. These findings revealed two overarching paradigms: conservation-driven binding, wherein antibodies target highly conserved residues crucial for viral function, and adaptability-driven binding, where antibodies exploit flexible or dynamic interfaces to maintain potency despite antigenic drift. In a recent study it was found that neutralizing effectiveness can be tied to allosteric effects as the ability to neutralize the virus is also linked to the quaternary changes it induces in the S protein trimer [52]. Differences in antibody-induced allosteric dynamics of the S protein can render weak, moderate, and strong neutralizing antibodies [52]. Collectively, structural and computational studies suggested that viral evolution is not merely a stochastic process but rather a finely tuned balance between immune evasion, receptor-binding affinity, and fitness constraints imposed by mutation-induced structural perturbations [53,54,55].

In the current study, we investigate the interplay between dynamic, energetic, and allosteric mechanisms that govern antibody binding and immune escape, focusing specifically on distinct groups of Class 4 antibodies. Class 4 antibodies represent a unique category of broadly reactive monoclonal antibodies that target a deeply conserved, cryptic epitope within the S-RBD protein. Here, we present a multi-dimensional analysis integrating structural mapping, conformational dynamics, mutational scanning, and binding free energy calculations, along with dynamic network modeling, to elucidate the molecular basis of antibody recognition, allostery, and immune escape across Class 4 group F1 (CR3022, EY6A, COVA1-16), group F2 (DH1047), and group F3 (S2X259) antibodies. We perform structural binding epitope mapping and analysis of closely-related cross-reactive but weakly neutralizing Class 4 group F1 antibodies CR3022 [31,56,57], EY6A [57], and COVA1-16 [58]. Through structural analysis we establish that in addition to CR3022, EY6A, and COVA1-16, Class 4 antibodies targeting the conserved cryptic site with similar binding footprints can be all classified as group F1 [26,27,37,38,39]. Additionally, we studied Class 4 antibodies DH1047 of group F2 [33], and S2X259 of group F3 [59]. The classification of RBD-directed antibodies has recently been redefined to include a larger set of antibodies and finer epitope binning [60]. To investigate dynamics, energetics, and binding profiles of these antibodies with S-RBD, we employed coarse-grained (CG) and atomistic molecular dynamics (MD) simulations, systematic mutational scanning of the antibody–RBD binding interfaces, and binding free energy calculations. Through network-based modeling of conformational ensembles, we explored the allosteric consequences of antibody binding. This work establishes a mechanistic continuum among Class 4 antibodies, from indirect allostery (group F1 antibodies) to hybrid (group F2 antibodies) and a combination of direct receptor blocking with localized allostery (group F3 antibodies), revealing key insights into how cryptic site recognition translates into functional impact. Importantly, it highlights that neutralization need not rely solely on physical occlusion of ACE2 but can emerge through dynamic reorganization of the RBD. These findings offer a useful computational framework for understanding antibody function beyond canonical epitopes and provide guidance for designing next-generation therapeutics and vaccines that harness the power of conformational dynamics and network-level control to combat evolving sarbecoviruses.

## 2. Materials and Methods

### 2.1. Coarse-Grained Molecular Simulations

The crystal and cryo-EM structures of the RBD–antibody complexes were obtained from the Protein Data Bank [61]. To efficiently explore the conformational landscape of the RBD–antibody complexes and generate diverse structural ensembles for subsequent atomistic simulations, we employed the CG-CABS approach [62,63,64,65,66,67]. The CABS-flex approach combines a high-resolution coarse-grained model and efficient search protocol capable of accurately reproducing all-atom MD simulation trajectories and dynamic profiles of large biomolecules on a long time scale [62,63,64,65,66,67]. In this CG model, the amino acid residues are represented by Cα, Cβ, the center of mass of side chains, and another pseudoatom placed in the center of the Cα-Cα pseudo-bond. The CABS-flex approach implemented as a Python 2.7 object-oriented standalone package was used in this study. Conformational sampling in the CABS-flex approach is conducted with the aid of Monte Carlo replica-exchange dynamics and involves local moves of individual amino acids in the protein structure and global moves of small fragments. The default settings were used in which soft native-like restraints are imposed only on pairs of residues fulfilling the following conditions: the distance between their *C*_α_ atoms is smaller than 8 Å, and both residues belong to the same secondary structure elements.

For each RBD–antibody complex, a total of 1000 independent CG-CABS simulations were performed. Each simulation consisted of 10,000 Monte Carlo cycles, with trajectory frames saved every 100 cycles. This extensive CG sampling protocol was designed to ensure thorough exploration of the conformational space accessible to each complex. To obtain high-resolution, all-atom trajectories for detailed analysis, we implemented a multi-scale strategy. From the final 5000 cycles (cycles 5000–10,000, considered the equilibrium phase) of each set of 1000 CG simulations, 100 representative conformations were selected at regular intervals for each complex. These conformations capture the diversity of the CG ensemble. Each selected CG conformation was then backmapped to an all-atom representation using the MODELLER-based reconstruction protocol provided by the CABS-flex package [68].

### 2.2. Molecular Dynamics Simulations

For all-atom MD simulations, the missing regions for the studied structures of the RBD–antibody complexes were reconstructed and optimized using the template-based loop prediction approach ArchPRED [69]. The side chain rotamers were refined and optimized by SCWRL4 tool [70]. The protonation states for all the titratable residues of the antibody and RBD proteins were predicted at pH 7.0 using Propka 3.1 software and web server [71,72]. The glycan chains were built using CHARMM-GUI Glycan Reader [73,74] and Modeller [68] at glycosylation sites N331 and N343 of RBDs.

The NAMD 2.13-multicore-CUDA package [75] with CHARMM36m force field [76] was employed to perform all-atom MD simulations for the RBD–antibody complexes. All-atom MD simulations were initiated from the 100 backmapped conformations generated for each RBD–antibody complex. Each system was solvated with TIP3P water molecules and neutralizing 0.15 M NaCl in a periodic box that extended 10 Å beyond any protein atom in the system [77]. All Na^+^ and Cl^−^ ions were placed at least 8 Å away from any protein atoms and from each other. MD simulations are typically performed in an aqueous environment in which the number of ions remains fixed for the duration of the simulation, with a minimally neutralizing ion environment or salt pairs to match the macroscopic salt concentration [78]. The heavy atoms in the complex were restrained using a force constant of 1000 kJ mol^−1^ nm^−1^ to perform the 500 ps equilibration simulation. Long-range, non-bonded van der Waals interactions were computed using an atom-based cutoff of 12 Å, with the switching function beginning at 10 Å and reaching zero at 14 Å. The SHAKE method was used to constrain all the bonds associated with hydrogen atoms. The simulations were run using a leap-frog integrator with a 2 fs integration time step. A 310 K temperature was maintained using the Nóse–Hoover thermostat with a 1.0 ps time constant, and 1 atm pressure was maintained using isotropic coupling to the Parrinello–Rahman barostat with a time constant of 5.0 ps [79,80]. The long-range electrostatic interactions were calculated using the particle mesh Ewald method [81] with a cut-off of 1.2 nm and a fourth-order (cubic) interpolation. The simulations were performed under an NPT ensemble with a Langevin thermostat and a Nosé–Hoover Langevin piston at 310 K and 1 atm. The damping coefficient (gamma) of the Langevin thermostat was 1/ps. In NAMD, the Nosé–Hoover Langevin piston method is a combination of the Nosé–Hoover constant pressure method [82] and piston fluctuation control implemented using Langevin dynamics [83]. An NPT production simulation was run on equilibrated structures for 1µs keeping the temperature at 310 K and a constant pressure (1 atm).

The combination of extensive CG sampling (1000 runs) followed by long atomistic simulations was designed to ensure convergence of conformational ensembles and the reliability of calculated observables such as RMSF, binding free energies, and dynamic network parameters. Convergence of key observables was assessed by analyzing the stability of calculated values across the ensemble of 100 atomistic trajectories per system. The consistency of results across this large number of independent simulations, combined with the extensive sampling within each trajectory, supports the robustness of our computed ensemble averages reported in this study.

### 2.3. Mutational Scanning Profiling

We conducted mutational scanning analysis of the binding epitope residues for the S RBD–antibody complexes. Each binding epitope residue was systematically mutated using all substitutions, and corresponding protein stability and binding free energy changes were computed. The BeAtMuSiC approach [84,85,86,87,88] was employed and evaluated the impact of mutations on both the strength of interactions at the protein–protein interface and the overall stability of the complex using statistical energy functions. BeAtMuSiC identifies a residue as part of the protein–protein interface if its solvent accessibility in the complex is at least 5% lower than its solvent accessibility in the individual protein partner(s). The binding free energy of the protein–protein complex can be expressed as the difference in the folding free energy of the complex and folding free energies of the two protein binding partners.

The change in the binding energy due to a mutation was calculated then as

(1) $$\Delta G_{bind}=G^{com}-G^{A}-G^{B}$$

$G^{com}$ is the free energy of the complex. This is the Gibbs free energy associated with the folded, bound state of the entire protein–protein complex (e.g., the Spike RBD–antibody complex). $G^{A}$ is the free energy of the first binding partner (e.g., the isolated Spike Receptor Binding Domain, RBD) in its unbound, folded state. $G^{B}$ is the free energy of the second binding partner (e.g., the isolated antibody) in its unbound, folded state.

The change in the binding energy due to a mutation was calculated then as

(2) $$\Delta \Delta G_{bind}=\Delta G_{bind}^{mut}-\Delta G_{bind}^{wt}$$

$\Delta \Delta G_{bind}$ is the change in binding free energy resulting from a specific mutation. This quantifies how the mutation affects the binding affinity compared to the wild-type (original) interaction. A positive *ΔΔG_bind* typically indicates weakened binding (the mutation makes binding less favorable or more difficult), while a negative *ΔΔG_bind* indicates strengthened binding (the mutation makes binding more favorable). $\Delta G_{bind}^{mut}$ is the binding free energy calculated using Equation (1), but for the mutated protein complex (e.g., a mutant RBD bound to the antibody). $\Delta G_{bind}^{wt}$ is the binding free energy calculated using Equation (1), but for the wild-type (unmutated) protein complex, serving as the reference state. We leveraged rapid calculations based on statistical potentials to compute the ensemble-averaged binding free energy changes using equilibrium samples from simulation trajectories. The binding free energy changes were obtained by averaging the results over 1000 and 10,000 equilibrium samples for each of the systems studied.

### 2.4. Binding Free Energy Computations

We calculated the ensemble-averaged changes in binding free energy using 1000 equilibrium samples obtained from simulation trajectories for each system under study. Initially, the binding free energies of the RBD–antibody complexes were assessed using the MM-GBSA approach [89,90]. Additionally, we conducted an energy decomposition analysis to evaluate the contribution of each amino acid during the binding of the RBD to antibodies [91,92]. The binding free energy for the RBD–antibody complex was obtained using:

(3) $$\Delta G_{\mathrm{bind}}=G_{\mathrm{RBD}-\mathrm{AB}}-G_{\mathrm{RBD}}-G_{\mathrm{AB}}$$

(4) $$\Delta G_{bind,MMGBSA}=\Delta E_{MM}+\Delta G_{sol}-T\Delta S$$

where *ΔE_MM_* is the total gas phase energy (sum of *ΔEinternal*, *ΔEelectrostatic*, and *ΔEvdw*), and *ΔGsol* is the sum of polar and non-polar contributions to solvation. Here, *G_RBD–ANTIBODY_* represents the average over the snapshots of a single trajectory of the complex, and *G_RBD_* and *G_ANTIBODY_* correspond to the free energy of the RBD and the antibody, respectively.

The polar and non-polar contributions to the solvation free energy were calculated using a Generalized Born solvent model and consideration of the solvent-accessible surface area [93]. MM-GBSA was employed to predict the binding free energy and decompose the free energy contributions to the binding free energy of a protein–protein complex on a per residue basis. The binding free energy with MM-GBSA was computed by averaging the results of computations over 1000 samples from the equilibrium ensembles. The standard error of the mean (SEM) for binding free energy estimates was calculated from the distribution of values obtained across the 1000 equilibrium snapshots sampled for each system.

In this study, we chose the “single-trajectory” protocol (one trajectory of the complex) because it is less noisy due to the cancellation of intermolecular energy contributions. Entropy calculations typically dominate the computational cost of MM-GBSA estimates. Therefore, it may be calculated only for a subset of the snapshots, or this term can be omitted [94,95]. In this study, the entropy contribution was not included in the calculations of binding free energies of the RBD–antibody complexes because the entropic differences in estimates of relative binding affinities were expected to be small owing to the small mutational changes and the preservation of the conformational dynamics [94,95]. MM-GBSA energies were evaluated with the MMPBSA.py script in the AmberTools21 package [96] and gmx_MMPBSA, a new tool to perform end-state free energy calculations from CHARMM and GROMACS trajectories [97].

### 2.5. Modeling of Residue Interaction Networks and Mutational Profiling of Allosteric Residue Centrality

To analyze protein structures, we employed a graph-based representation where residues are modeled as network nodes, and non-covalent interactions between residue side-chains define the edges [98,99]. This approach captures the spatial and functional relationships between residues, providing insights into the protein structural and dynamic properties. The Residue Interaction Network Generator (RING) program [100,101,102] was used to generate the initial residue interaction networks from the crystal structures of the protein complexes. We computed the network parameters, such as shortest paths and betweenness centrality, to identify residues critical for communication within the protein structure. The short path betweenness (SPC) of residue *i* is defined to be the sum of the fraction of shortest paths between all pairs of residues that pass through residue *i*:

(5) $$C_{b}(n_{i})=\sum_{j<k}^{N}\frac{g_{jk}(i)}{g_{jk}}$$

where $g_{jk}$ denotes the number of shortest geodesics paths connecting j and *k*, and $g_{jk}(i)$ is the number of shortest paths between residues j and *k* passing through the node $n_{i}$. Residues with high occurrence in the shortest paths connecting all residue pairs have a higher betweenness values. Residues with high betweenness centrality values were identified as key mediators of communication within the protein structure network. All parameters were computed using the Python package NetworkX (NetworkX 3.5, released in 2025) [103] and the Cytoscape (Cytoscape 3.10.3 released in 2024.) package for network analysis [104,105,106].

Through mutation-based perturbations of protein residues, we computed dynamic couplings of residues and changes in SPC averaged over all possible modifications in a given position. The change in SPC upon mutational changes of each node is reminiscent of the calculation of residue centralities by systematically removing nodes from the network.

(6) $${\Delta L}_{i}=\langle {\left|\left|\Delta L_{i}^{node}\left(j\right)\right|\right|}^{2}\rangle$$

where *i* is a given site, *j* is a mutation, and <⋯> denotes averaging over mutations. $\Delta L_{i}^{node}\left(j\right)$ describes the change in SPC parameters upon mutation j in a residue node i. $\Delta L_{i}$ is the average change of ASPL triggered by mutational changes in position i. The Z-score was then calculated for each node as follows:

(7) $$Z_{i}=\frac{\Delta L_{i}-\langle \Delta L\rangle }{\sigma }$$

$\langle \Delta L\rangle$ is the change in the SPC network parameter under mutational scanning averaged over all protein residues and σ is the corresponding standard deviation. The ensemble-average Z score changes were computed from network analysis of the conformational ensembles of antibody–RBD complexes using 1000 snapshots of the simulation trajectory.

## 3. Results

### 3.1. Structural Analysis of the RBD Complexes with Class 4 Antibodies

**Figure 1:**
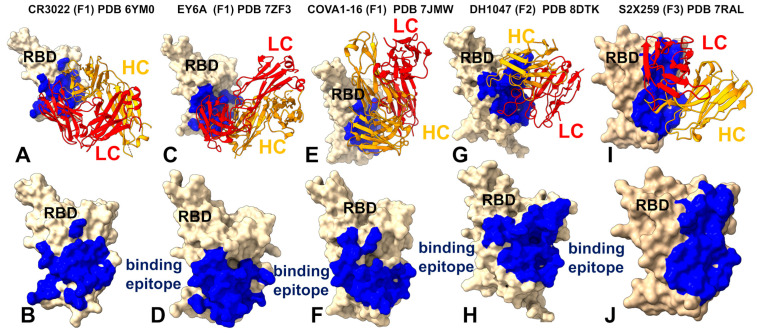
Structural organization of the RBD complexes and binding epitopes for Class 4 antibodies. (**A**) The structure of Class 4, group F1 CR3022 with RBD (pdb id 6YM0). The RBD is represented with wheat-colored surface, the heavy chain with orange ribbons and the light chain with red ribbons. (**B**) The RBD and binding epitope footprint for CR3022. The binding epitope residues are shown with blue surface. (**C**) The structure of Class 4 group F1 antibody EY6A bound with RBD (pdb id 7ZF3). (**D**) The RBD and binding epitope footprint for EY6A. (**E**) The structure of Class 4 group F1 antibody COVA1-16 bound with RBD (pdb id 7JMW). (**F**) The RBD and binding epitope footprint for COVA1-16. (**G**) The structure of Class 4 group F2 antibody DH1047 bound with RBD (pdb id 8DTK). (**H**) The RBD and binding epitope footprint for DH1047. (**I**) The structure of Class 4 group F3 antibody S2X259 bound with RBD (pdb id 7RAL). (**J**) The RBD and binding epitope footprint for S2X259.

**Figure 2:**
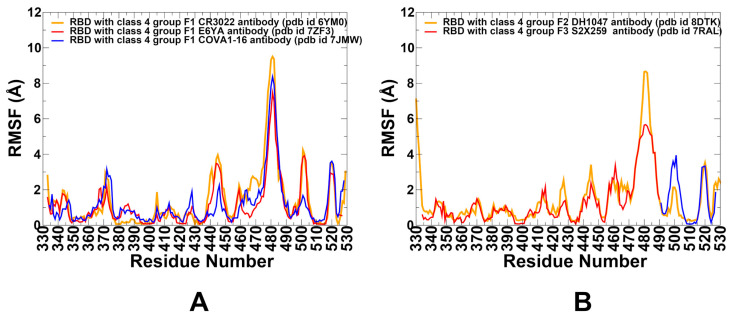
RMSF profiles of RBD residues upon binding with Class 4 antibodies. (**A**) RMSF profiles for residues of the RBD in complexes with group F1 Class 4 antibodies. The figure compares the dynamic behavior of the RBD upon binding to CR3022 (PDB ID: 6YM0, orange), EY6A (PDB ID: 7ZF3, red), and COVA1-16 (PDB ID: 7JMW, blue). Residues within the central β-sheet structure (e.g., residues 354–358, 376–379, 394–403, 432–437, 452–454, 492–494, and 507–516) exhibit low RMSF values across all three antibodies, indicating minimal flexibility. Residues ~470–490 show elevated RMSF values, particularly for group F1 antibodies, reflecting enhanced local flexibility in this region. (**B**) RMSF profiles for residues of the RBD in complexes with group F2 (DH1047) and group F3 (S2X259) Class 4 antibodies. Group F2 (DH1047, PDB ID: 8DTK, orange): residues 450–470 exhibit moderately increased mobility, while the 470–490 loop shows reduced flexibility compared to group F1 antibodies. Group F3 (S2X259, PDB ID: 7RAL, blue): S2X259 also displays reduced flexibility in the 470–490 loop, consistent with its more direct engagement of the ACE2 interface.

We first performed structural binding epitope mapping and analysis of closely-related cross-reactive but weakly neutralizing Class 4 antibodies CR3022 (group F1, Class 4) [31,56,57], EY6A [57], and COVA1-16 [58] (Figure 1, Supplementary Materials, Tables S1–S3). The CR3022 epitope is typically only accessible when at least two RBDs of the S protein are in the “up” conformation (Figure 1A,B). EY6A and CR3022 bind to the same epitope on the RBD but EY6A binds at about 70 degrees to the perpendicular axis of the α3-helix, which is central to both epitopes (Figure 1C,D). Both CR3022 and EY6A target residues 368–392 on the RBD, CR3022 interacts with sites 408, 427–433, and 515–519, while EY6A makes contacts with 411–414 and 427–430 (Figure 1 and Figure 2). The binding epitope for COVA1-16 showed a similar footprint of residues 368–385, 408, 412–415, and 427–430 (Figure 1 and Figure 2). Structural mapping of the binding epitopes for these three antibodies highlighted the same mode of binding and small antibody-specific variations around the cryptic site (Figure 1A–F). It is evident that CR3022 makes contacts with more residues in the binding site, particularly residues 515–519 (Figure 1A,B). However, our analysis strongly suggests that in addition to CR3022, both EY6A and COVA1-16 are Class 4 antibodies that can be attributed to the same group F1 (Figure 1C–F).

Members of the F1 group, such as CR3022, EY6A, and COVA1-16, require a wide-open RBD to engage and do not directly block ACE2, therefore displaying weak neutralizing activities. The DH1047 antibody interacts with specific regions of the RBD, including those known to be mobile, through its heavy-chain complementarity-determining region 3 (HCDR3) and light-chain complementarity-determining region 1 (LCDR1) and LCDR3 (Figure 1G,H). By directly engaging with these areas, the antibody can restrict their movement. Structural analysis reveals that the binding of DH1047 to the RBD creates steric hindrance and reaches towards the so-called “right shoulder” of the RBD [107], making some partial overlap of the ACE2 binding site. The notable extension of the binding epitope for group F2 DH1047 is formation of multiple contacts with R408 and additional interactions with residues 500–508 that overlap with the ACE2 binding site (Figure 1G,H, Supplementary Materials, Table S4). For group F3 S2X259, we observe even further movement of the epitope to the right shoulder, towards the ACE2 binding site, making contacts with residues 377–385, 404–408, and 501–508 (Figure 1I,J, Supplementary Materials, Table S5). Interestingly, the key interacting sites D405, R408, V503, G504, and Y508 for group F3 S2X259 (Figure 1I,J, Supplementary Materials, Table S5) can enable interference with ACE2 but also are associated with the major escape sites for this group of antibodies [26,27,37,38]. This analysis emphasizes the dual nature of the S2X259 epitope where there is a central, conserved core which is arguably resistant to mutational drift due to its functional importance, flanked by more flexible peripheral regions that may accommodate escape mutations under selective pressure.

We also illustrated the overlap in the binding epitope residues for CR3022 of group F1, DH1047 of group F2, and S2X259 of group F3 (Supplementary Materials, Figure S1). The Venn diagram for group F1 antibodies (Supplementary Materials, Figure S1A) reveals that CR3022, EY6A, and COVA1-16 share a significant number of binding hotspots, indicating a common mode of recognition centered around the deeply conserved hydrophobic core of the RBD. The shared residues (~15%) highlight the conservation of epitope targeting within this group, which explains their broad reactivity across sarbecoviruses. However, each antibody exhibits unique features. CR3022 engages more residues in the binding site, suggesting a slightly broader footprint compared to EY6A and COVA1-16. EY6A binds at a similar region but with a slightly different orientation, leading to subtle differences in contact geometry and mutational sensitivity. It is notable from structural mapping of the epitopes that group F2 DH1047 has a significant overlap with group F1 antibodies but begins to extend partly towards the ACE2 binding interface (Supplementary Materials, Figure S1B).

### 3.2. Conformational Dynamics of the RBD Complexes with Antibodies Using Coarse-Grained and Atomistic Simulations

We performed multiple CG-CABS and atomistic simulations of the RBD–antibody complexes. The root-mean-square fluctuation (RMSF) profiles provide a detailed view of the dynamic behavior of RBD residues upon antibody binding, highlighting both shared features and notable differences among the antibodies. These profiles reveal both shared characteristics and particular features for each antibody (Figure 2A). This central β-sheet core (β1: residues 354–358; β2: residues 376–379; β3: residues 394–403; β4: residues 432–437; β5: residues 452–454; β6: residues 492–494; β7: residues 507–516) and α-helices exhibit low RMSF values across all Class 4 antibodies, indicating minimal flexibility in these regions.

Conformational dynamics analysis reveals that group F1 antibodies engage a deeply conserved, cryptic epitope located in the core of the RBD, accessible only when at least two RBDs adopt the “up” conformation. These antibodies stabilize the central β-sheet structure of the RBD—composed of residues such as β1–β7—reducing local flexibility and reinforcing structural integrity. However, their binding also increases mobility in the receptor-binding motif (RBM) loop (residues ~470–490), promoting greater conformational sampling and facilitating access to the cryptic site. This redistribution of rigidity and flexibility appears to underpin their neutralization mechanism: rather than directly blocking ACE2, they modulate the RBD conformational equilibrium, indirectly impairing receptor engagement by favoring non-productive open states. One of the most striking findings from this comparative analysis is the common effect these antibodies have on RBD flexibility. The data consistently show that antibody binding stabilizes the rigid RBD core while increasing mobility in the RBM loop region. The increased conformational diversity in this region may disrupt the precise alignment required for efficient ACE2 binding, subtly altering the energetic landscape without direct competition. This mechanism aligns well with experimental observations from surface plasmon resonance (SPR) and bio-layer interferometry (BLI) studies showing that CR3022 binding slows ACE2 association and accelerates dissociation via allosteric modulation rather than physical occlusion [57].

Group F2 antibody DH1047 exhibits a more direct influence on the ACE2 interface compared to F1 antibodies, while still engaging the conserved RBD core. RMSF analysis shows minor increases in flexibility in residues 450–470, coupled with reduced mobility in the RBM-containing loop (~470–490), indicating a shift in dynamic control toward stabilizing the ACE2-contacting regions (Figure 2B). This shift suggests stabilization of regions closer to the ACE2 interface, supporting a hybrid mode of action that combines allosteric effects with partial steric hindrance, resulting in higher potency but increased escape vulnerability.

The group F3 antibody S2X259 demonstrates the most pronounced impact on RBD dynamics, reflecting its direct competition with ACE2. RMSF profiles show significantly reduced flexibility in the RBM loop (~470–490), consistent with strong binding that locks the RBD into a conformation incompatible with efficient ACE2 engagement (Figure 2B). These shifts suggest that both DH1047 and S2X259 modulate RBD dynamics in distinct ways, subtly reshaping the conformational landscape to enhance direct competition with ACE2.

**Figure 3:**
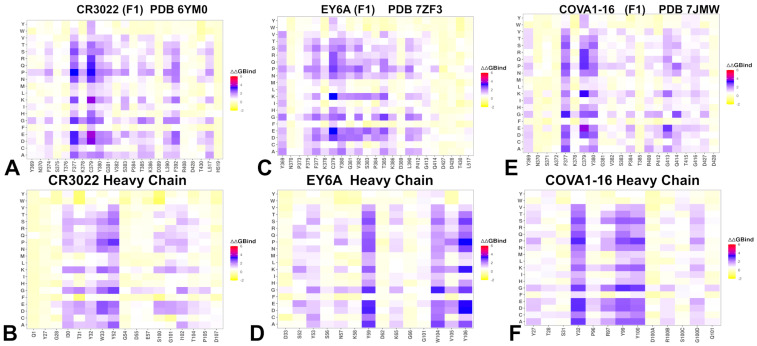
Ensemble-based dynamic mutational profiling of the RBD intermolecular interfaces in the RBD complexes. Mutational heatmaps for RBD complex with group F1 CR3022 antibody (**A**,**B**), the RBD complex with group F1 E6YA antibody (**C**,**D**), and the RBD complex with group F1 COVA1-16 antibody (**E**,**F**). The mutational scanning heatmaps are shown for the interfacial RBD residues and interfacial heavy chain residues of respective Class 4 group F1 antibodies. The heatmaps show the computed binding free energy changes (in kcal/mol) for 20 single mutations of the interfacial positions. The standard errors of the mean for binding free energy changes using randomly selected 1000 conformational samples (0.06–0.12 kcal/mol) obtained from the atomistic trajectories.

To sum up, the RMSF profiles reveal a progressive shift in conformational dynamics across Class 4 antibodies from groups F1 to F3. Group F1 antibodies stabilize the core while increasing flexibility in the RBM loop, promoting allosteric interference with ACE2 binding (Figure 3, Table 1). In contrast, groups F2 and F3 exhibit reduced flexibility in the RBM loop, reflecting their closer proximity to the ACE2 interface and stronger interactions with critical residues involved in receptor binding (Table 1). These results may underscore the mechanistic evolution of neutralization strategies among Class 4 antibodies, transitioning from indirect allostery (F1) to partial steric hindrance (F2) and direct competition with ACE2 (F3).

### 3.3. Mutational Profiling of Antibody–RBD Binding Interaction Interfaces Reveals Molecular Determinants of Immune Sensitivity and Emergence of Convergent Escape Hotspots

Using the conformational ensembles of the RBD–antibody complexes, we performed mutational analysis of the S protein binding with antibodies. Mutational scanning of RBD–antibody complexes provided insight into the epitope sensitivity and escape vulnerability of Class 4 group F1 antibodies—CR3022, EY6A, and COVA1-16. Mutational profiling of CR3022 binding identified a set of key interface residues that are essential for antibody recognition. F377, C379, Y380, and T385 emerged as dominant hotspots (Figure 3A). Additional contributions come from K378, G381, V382, S383, P384, L390, and F392, which also play supportive roles in maintaining epitope integrity. Our results are consistent with the DMS data revealing that residues 383–386, 390, and 392 represent major escape hotspots for CR3022 and other F1-class antibodies. These residues lie at the edge of the epitope, suggesting that immune pressure drives mutations here to evade antibody recognition without compromising viral function. EY6A binds to a nearly identical region on the RBD as CR3022 (Figure 1 and Figure 2) yet exhibits a somewhat distinct mutational sensitivity profile reflecting differences in orientation and contact geometry. The heavy chain of CR3022 features hotpots in positions I30, T31, Y32, and W33 (Figure 3B).

Mutational profiling revealed that EY6A engages similar dominant residues—including F377, C379, Y380, T385, and S383—confirming that both antibodies recognize a shared structural motif. However, EY6A also makes weaker interactions with P412 and G413, which are located slightly further from the central hotspot (Figure 3C). This broader interaction profile may offer modest resilience to certain escape mutations, although it remains sensitive to changes at key peripheral sites. The heavy chain of EY6A shows strong hotspot contributions from Y59, W104, V105, and Y106, underscoring the importance of aromatic stacking and van der Waals forces in stabilizing this complex (Figure 3D).

For COVA1-16, mutational analysis confirms a binding mode overlapping with CR3022 and EY6A, targeting residues 368–385, 408, 412–415, and 427–430, with major contributions from F377, K378, C379, and Y380 (Figure 3E). Minor hotspots such as R408 and G413 were also identified, suggesting that COVA1-16 extends its influence closer to the ACE2 interface than CR3022 or EY6A. Despite this, it retains the hallmark features of group F1 antibodies—broad reactivity due to targeting a structurally conserved RBD core, and vulnerability to mutations affecting epitope integrity or solvent exposure. Its heavy chain interactions are dominated by Y32, Y99, and Y100, highlighting a unique residue-level specificity that may shape its conformational adaptability and dynamic coupling with the RBD (Figure 3F). Structural epitope mapping of group F1 Class 4 antibodies CR3022, EY6A, and COVA1-16 highlighted the fact that while all three antibodies target a deeply conserved hydrophobic core within the RBD, there are subtle differences in their contact footprints and residue-specific interactions, reflecting minor variations in orientation and binding geometry (Supplementary Materials, Figure S2).

Taken together, these mutational scanning results highlight a trade-off between conservation and conformational dependency in group F1 antibodies. While their targeting of a deeply conserved RBD core ensures broad sarbecovirus reactivity, their reliance on rare “up” conformations and sensitivity to edge mutations limit their neutralization potency, especially against evolving variants.

**Figure 4:**
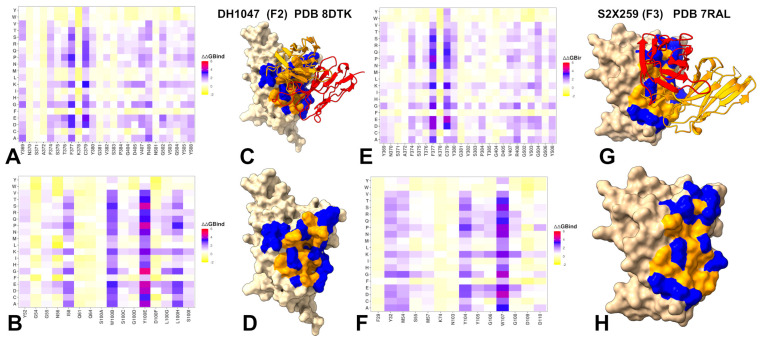
Mutational heatmaps and epitope mapping of groups F2 and F3 of Class 4 antibodies binding to the RBD. Mutational heatmaps of the RBD binding interface residues for RBD complex with group F2 DH1047 antibody (**A**) and mutational heatmap of the heavy chain of DH1047 (**B**). The three-dimensional structures of the DH1047 complex with RBD (**C**), and a detailed view of the RBD, the epitope, and binding hotspots for DH1047 (**D**) (PDB ID: 8DTK). Mutational heatmaps of the RBD binding interface residues for RBD complex with group F3 S2X259 antibody (**E**) and mutational heatmap of the heavy chain of S2X259 (**F**). The three-dimensional structures of the S2X259 complex with RBD (**G**), and a detailed view of the RBD, the epitope, and binding hotspots for S2X259 (**H**) (PDB ID: 7RAL). The RBD is represented with wheat-colored surface. The epitope sites are highlighted with blue surface and binding hotspots with orange surface.

The mutational scanning analysis of group F2 antibody DH1047 and group F3 antibody S2X259 provides key insights into how these Class 4 antibodies engage the RBD and respond to immune-evading mutations (Figure 4). While both target epitopes that overlap with the ACE2 interface, they do so with distinct energetic and functional consequences. For DH1047, mutational profiling identifies a set of critical RBD residues—including Y369, F374, T376, F377, C379, Y380, R408, V503, Y505, and Y508—that are essential for antibody recognition. Among these, T376, R408, V503, and Y508 emerge as major destabilization sites, where single-point mutations such as T376A (ΔΔG = 2.15 kcal/mol), R408A/S (ΔΔG = 2.35–2.44 kcal/mol), and Y508H/N significantly impair binding (Figure 4A). The emergence of these mutations highlights a growing escape risk associated with proximity to the ACE2 interface, despite the functional constraints on many of these residues. These findings indicate that even though the core RBD region remains conserved, certain peripheral residues under immune pressure are mutable, especially those near the ACE2-binding motif. Importantly, the heavy chain residue Y100D in DH1047 forms extensive hydrogen bonds with S371, T376, F377, and Y369, reinforcing the idea that this region plays a critical role in stabilizing the antibody–RBD complex (Figure 4B). However, the presence of Omicron BA.1/BA.2 mutations (S371L, S373P, S375F) indirectly compromises binding by altering the local conformation or solvent exposure of nearby hotspot residues, further highlighting the network-level sensitivity of DH1047 to changes in the RBD landscape. Structural mapping of the DH1047 complex with RBD and the footprint of the binding hotspots on the RBD (Figure 4C,D) illustrates a broadly distributed allocation of the binding hotspots. Notably the partial overlap with the ACE2 binding site does not involve strong binding hotspots, suggesting that these interactions can modulate the binding and immune escape patterns for this group of antibodies.

Group F3 antibody S2X259 engages the RBD through an even more direct overlap with the ACE2 footprint, interacting strongly with D405, R408, V503, G504, and Y508—all of which are key contact points for host receptor engagement (Figure 4E,F). This proximity enables direct competition with ACE2, resulting in stronger neutralization potency, but also increases susceptibility to immune escape. Mutational data show that substitutions such as D405N, R408S, and T376A severely disrupt binding, consistent with their high energetic contributions to the interaction (Figure 4E). R408 and T376 remain mutable under selective pressure, making them high-risk escape positions. The emergence of T376A and R408S in Omicron BA.2 exemplifies how immune pressure drives mutations at the periphery of functionally constrained regions, compromising antibody binding while preserving viral fitness [26,27,37,38]. Group F3 antibody S2X259 exhibits the highest neutralization potency due to direct competition but faces the greatest escape risk, demonstrating the trade-off between potency and durability as antibodies evolve closer to the ACE2 interface.

To conclude, mutational profiling analysis of Class 4 antibody–RBD interactions showed that while all antibodies target a conserved RBD core, distinct differences emerge in their escape vulnerabilities. F1 antibodies target the deeply conserved core but are vulnerable to mutations at epitope edges (e.g., 383–386, 390, 392 for CR3022), limiting their potency. F2 antibody DH1047 exhibits a hybrid profile, engaging residues closer to the ACE2 interface (T376, R408, V503, Y508), increasing potency but also escape risk, with key mutations like T376A and R408S significantly impairing binding. F3 antibody S2X259 shows the most direct overlap with ACE2, interacting strongly with critical receptor-binding residues (D405, R408, V503, G504, Y508), resulting in the highest potency but greatest escape susceptibility. The analysis reveals an evolutionary trend: as Class 4 antibodies shift from indirect allostery (F1) towards direct steric competition (F3), their neutralization strength increases, but so does their vulnerability to immune escape, highlighting a fundamental trade-off between potency and durability.

### 3.4. MM-GBSA Analysis of the Binding Energetics for Class 4 Antibody Complexes

The MM-GBSA analysis provides a detailed energetic dissection of how Class 4 antibodies engage the RBD, revealing distinct interaction patterns that reflect their neutralization mechanisms and escape vulnerabilities. By decomposing the total binding free energy into van der Waals (VDW) and electrostatic (ELE) components, we gain insight into the molecular determinants of binding affinity and how mutations can disrupt these interactions, leading to immune evasion.

For F1 antibodies, MM-GBSA results highlight a strong reliance on hydrophobic interactions, particularly involving the conserved core residues 377–382, which form a tightly packed interface critical for complex stability (Supplementary Materials, Figure S3A,B). These interactions, centered on the RBD’s central β-sheet, underpin the broad sarbecovirus reactivity of this group. Electrostatic contributions are modest and distributed, with residues like K378 and D428 providing fine-tuning rather than dominant stabilization (Supplementary Materials, Figure S3C). EY6A binds to a similar region of the RBD as CR3022, yet its energetic profile reveals some specific features. The central core residues 378–385 are the main contributors to binding, with favorable van der Waals interactions playing a dominant role (Supplementary Materials, Figure S3D,E). EY6A engages a slightly broader region than CR3022, including residues P412 and G413, which may offer some resilience against localized mutations (Supplementary Materials, Figure S3D,E). While K378, K386, D405, D427, and D428 exhibit favorable electrostatic contacts (Supplementary Materials, Figure S3F), these interactions are largely offset by unfavorable solvation penalties, resulting in only marginal net stabilization of the complex. This suggests that EY6A relies more heavily on hydrophobic interactions than on electrostatic complementarity, making it potentially more robust against certain polar substitutions, but vulnerable to mutations that alter hydrophobic packing including those seen in Omicron variants. COVA1-16 presents an energy profile marked by a more distributed pattern of hotspot residues compared to CR3022 and EY6A. Key contributors include C379, Y369, F377, Y380, and P384, along with Y380, D427, and R408, emphasizing the importance of aromatic stacking and aliphatic interactions in stabilizing the complex (Supplementary Materials, Figure S3G,H). Electrostatically, favorable contributions arise from D420, D427, and D428, reinforcing the idea that charged residues play a supportive but secondary role in binding (Supplementary Materials, Figure S3I). This broader footprint may confer some resilience to localized mutations, although it also increases the number of potential escape pathways, particularly at positions 412–415 and 427–428, which are under growing immune pressure. Importantly, the interactions of COVA1-16 extend slightly toward residues partly overlapping with the ACE2 interface, suggesting that its neutralization mechanism may involve a mild competitive component, despite being classified as a Class 4 antibody. SEM were calculated from the ensemble of snapshots used in the MM-GBSA analysis (*n* = 1000 samples). The SEM values for the binding free energies components for group F1 antibodies range from 0.12 to 0.18 kcal/mol for the interfacial RBD residues and 0.08 to 0.15 kcal/mol for the interfacial heavy chain residues, reflecting the variability in energy calculations across the sampled conformational ensemble. One of the most striking insights from this analysis is the delicate interplay between different energetic components that define the binding landscape of group F1 antibodies. In CR3022 and EY6A, hydrophobic interactions dominate, contributing to high conservation and broad reactivity, but also rendering them vulnerable to mutations that destabilize local structure. In contrast, a somewhat wider hotspot distribution and greater involvement of electrostatics found for the COVA1-16 antibody suggest a more adaptable binding mode, though one that may be more sensitive to polar or charged mutations. The relative energetic contributions calculated by MM-GBSA show excellent qualitative agreement with experimentally determined escape mutation profiles from DMS [26,27], validating the method’s ability to identify key hotspot residues.

The strongest binding hotspots for group F2 DH1047 include Y505, R408, V503, T375, F377, K378, Y380, C379, T415, and F374 (Supplementary Materials, Figure S4A). These residues form a structurally conserved yet partially exposed interface, highlighting the delicate balance between conservation and accessibility. For this group F2 antibody, residues V503, Y505, T376, S375, Y380, F377, and R408 dominate the hydrophobic contacts, providing the primary stabilizing force for the complex (Supplementary Materials, Figure S4B). While K378, R408, and K386 contribute favorable electrostatics, these interactions are offset by solvation penalties, resulting in only marginal net stabilization (Supplementary Materials, Figure S4C). Notably, DH1047 exhibits a hybrid interaction pattern, combining elements of both group F1 conservation and group F3 interference with ACE2 binding. This suggests that group F2 antibodies may occupy an evolutionary intermediate stage, where increased potency comes at the cost of greater sensitivity to immune pressure, especially in evolving variants.

The group F3 S2X259 antibody exhibits a binding mode that extends even closer to the ACE2 interface, which may effectively transition mechanistically from allosteric effects on ACE2 binding toward direct competitive inhibition. We found that residues V503, R408, P384, F377, and D405 form robust hydrophobic interactions, anchoring the antibody firmly to the RBD (Supplementary Materials, Figure S4D,E). The favorable electrostatic interactions are observed at R408, K378, R403, D389, and K417, further stabilizing the complex (Supplementary Materials, Figure S4F). The combination of strong hydrophobic and electrostatic interactions creates a robust binding interface that overlaps significantly with the ACE2 footprint, particularly at R408, V503, and G502, which are among the most critical residues for viral entry. The MM-GBSA results suggest that the neutralization potency of S2X259 stems from its ability to engage both hydrophobic cores and charged interfaces, thereby combining structural conservation with functional interference. For group F2 and F3 antibodies, the SEM values for RBD residues were within 0.08–0.15 kcal/mol.

When comparing the energetic profiles and escape vulnerabilities across group F1 (CR3022, EY6A, COVA1-16), group F2 (DH1047), and group F3 (S2X259), an interesting evolutionary trajectory emerges reflecting a progression from deeply cryptic, allosteric engagement (F1), through partial overlap with ACE2 and enhanced hydrophobic/electrostatic synergy (F2), to direct receptor competition with strong energetic coupling (F3). From this comparative view, we observe a gradual shift in energetic strategy. Group F1 antibodies rely on deeply conserved hydrophobic cores, offering broad reactivity but low potency due to low accessibility and indirect neutralization mechanisms. Group F2 antibodies begin to leverage electrostatic complementarity, enhancing affinity and partial receptor blocking, but at the cost of increased escape susceptibility. Group F3 antibodies achieve the strongest binding synergy, combining hydrophobic stability with electrostatic reinforcement, allowing them to effectively compete with ACE2—albeit with greater vulnerability to mutations at key receptor-contacting residues.

### 3.5. Exploring Allosteric Binding Pathways Using Dynamic Network Analysis

**Figure 5:**
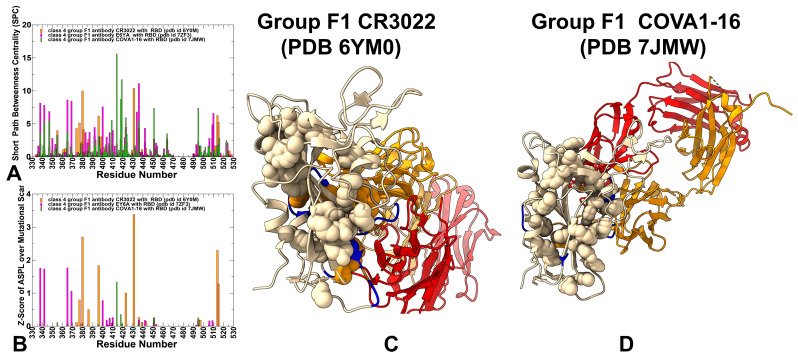
The ensemble-averaged SPC centrality (**A**) and the average Z-score of ASPL over mutational scan (**B**) for the RBD residues for Class 4 group F1 antibody complexes. CR3022 with RBD, pdb id 6YM0 (orange filled bars), EY6A with RBD, pdb id 7ZF3 (magenta filled bars), and COVA1-16 with RBD, pdb id 7JMW (green filled bars). (**C**) Structural mapping of allosteric network centers for Class 4 group F1 CR3022 antibody with RBD. (**D**) Structural mapping of allosteric network sites for Class 4 group F1 COVA1-16 antibody with RBD. The heavy chain is represented with orange ribbons, the light chain with red ribbons. The binding epitope residues are shown with blue surface, while binding hotspots are in orange and the allosteric residue interaction network with high SPC and Z-score ASPL values is represented with wheat-colored spheres.

Through ensemble-based averaging over mutation-induced changes in the network parameters we identified positions in which mutations on average cause network changes. Allosteric hotspots are identified as residues in which mutations incur significant perturbations of the global residue interaction network that disrupt the network connectivity and cause a significant impairment of network communications and compromise signaling. We first analyzed the distribution of the SPC and Z-score ASPL parameter for Class 4 group F1 CR3022, EY6A, and COVA1-16 antibodies (Figure 5A,B). Across all three antibodies, the central RBD core emerges as a highly connected hub in the residue interaction network. For CR3022, high-centrality residues include 431, 380, 396, 377, 423, 436, 374, 381, 355, 392, and 398—many of which are deeply conserved and structurally critical. These positions form a rigid scaffold that underpins the overall stability of the RBD and serves as a conduit for allosteric signals originating from the cryptic epitope. Similarly, EY6A and COVA1-16 also highlight core residues as key mediators of network connectivity. EY6A engages residues such as 365, 338, 342, 369, 400, 410, and 407, while COVA1-16 identifies 414, 418, 355, 436, 396, 341, 338, 379, 393, and 397 as high-centrality nodes (Figure 5A,B). Notably, several of these residues overlap with those highlighted by CR3022, reinforcing the idea that a shared structural framework exists across group F1 antibodies, even if each antibody approaches it with slight variations in orientation or contact profile. Importantly, all three antibodies appear to influence residues near the ACE2 interface, including 451, 495, 497, 439, 443, and 506, through indirect allosteric coupling rather than direct steric interference. This suggests that despite not directly overlapping with the ACE2-binding motif, group F1 antibodies can still exert functional consequences on receptor engagement, mediated via long-range dynamic reorganization of the RBD. Structural mapping of the high centrality sites in the CR3022 complex (Figure 5C) and COVA1-16 complex (Figure 5D) displayed a dense interaction network that produced rigidification of the RBD central core engaging “the right shoulder” of the RBD [107,108].

While the overall dynamic footprint and network topology are broadly similar across the three antibodies, minor differences emerge in the specific residues involved and their relative contributions to network integrity. EY6A shows notable involvement of residues 365, 338, 342, and 407, which are slightly more peripheral compared to the CR3022-centric core. It also interacts more strongly with positions near the ACE2 interface, such as 495, 453, 493, and 506, potentially enhancing its modulatory influence over receptor-binding dynamics. COVA1-16, in contrast, engages a broader set of core residues, including 414, 418, 393, and 397, which may reflect a more distributed mode of network interaction. Its mutational sensitivity profile includes residues at the edge of the RBM, such as 406 and 493, suggesting that COVA1-16 may be more sensitive to mutations affecting the transition between open and closed RBD states. Despite these small variations, the overall architecture of the allosteric network remains highly conserved, supporting the hypothesis that group F1 antibodies operate through a common functional mechanism, albeit with fine-tuned differences in network coupling and residue-specific interactions. Hence, the network-based allosteric analysis reveals that group F1 Class 4 antibodies leverage a modular and evolutionarily preserved interaction framework. Their neutralization strategy relies on dynamic modulation rather than direct steric blocking, reshaping the conformational ensemble of the RBD to disfavor ACE2 engagement. While this mechanism provides robustness against escape at core residues, it also introduces vulnerabilities at the periphery of the binding epitopes. Each antibody engages a distinct subset of network hubs, yet all converge on a common dynamic outcome—interference with the efficiency of ACE2 engagement through conformational and kinetic modulation. The subtle differences in residue-level network contributions suggest that each antibody may fine-tune the allosteric signal in distinct ways, possibly influencing their susceptibility to escape mutations or their efficacy in different spike conformations.

**Figure 6:**
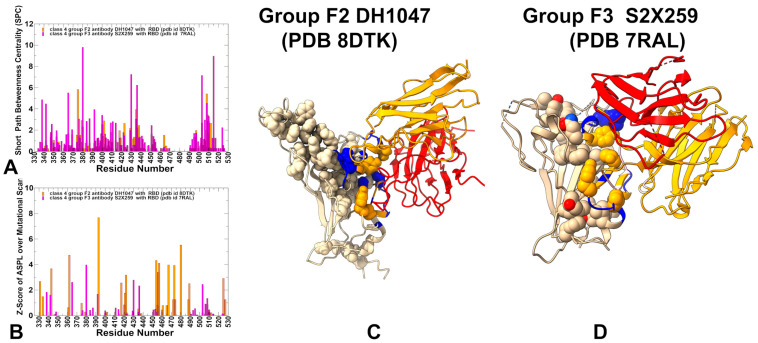
The ensemble-average SPC centrality (**A**) and the average Z-score of ASPL over mutational scan (**B**) for the RBD residues for Class 4 group F2 antibody complexes. DH1047 with RBD, pdb id 8DTK (orange filled bars) and group F3 antibody complex S2X259 with RBD, pdb id 7RAL (magenta filled bars). (**C**) Structural mapping of allosteric network sites for Class 4 group F2 antibody complex DH1047 with RBD, pdb id 8DTK. (**D**) Structural mapping of allosteric network sites for the Class 4 group F3 antibody complex S2X259 with RBD, pdb id 7RAL. The heavy chain is shown with orange ribbons, the light chain with red ribbons. The binding epitope residues are shown with blue surface, while binding hotspots are in orange and the allosteric residue interaction network with high SPC and Z-score ASPL values is represented with wheat-colored spheres.

Network analysis of group F2 antibody DH1047 revealed redistribution of the residue interaction network, with increasing localization around key positions involved in receptor binding. The high centrality residues identified for DH1047 include RBD ore elements (residues 393, 480, 362, 343, 331) as well as some ACE2-proximal residues (453, 454, 456, 457, 503, 507, 508) (Figure 6A,B). This dual involvement suggests that DH1047 engages both the conserved structural scaffold and functionally relevant regions, allowing it to exert both global conformational effects and local interference with ACE2 engagement. Structural mapping further supports this hybrid mechanism, showing that the allosteric signal now propagates more directly from the cryptic site toward the ACE2-binding motif, forming a more focused communication pathway compared to group F1 antibodies (Figure 6C). However, this increased proximity to the ACE2 interface also introduces new escape vulnerabilities, particularly at T376, K378, and R408, where mutations such as T376A and R408S can severely compromise binding. These findings highlight a trade-off between enhanced potency and increased sensitivity to antigenic drift, positioning group F2 antibodies as an intermediate stage in the mechanistic evolution of Class 4 antibodies.

Group F3 antibody S2X259 represents the most advanced adaptation in this trajectory, engaging the RBD through a highly localized network that overlaps extensively with the ACE2 footprint with the high-centrality positions (453, 454, 457, 467, 493) that overlap with the ACE2 binding interface (Figure 6A,B). S2X259 forms more localized and direct communication pathways from the cryptic site to the receptor interface (Figure 6D), enabling dual modes of action: direct steric interference with ACE2 via overlapping contacts and allosteric reinforcement by stabilizing RBD conformations incompatible with efficient receptor engagement.

In summary, the dynamic network analysis reveals a clear evolutionary progression among Class 4 antibodies, marked by increasingly focused allosteric signaling pathways that link the cryptic epitope—located deep within the RBD core—to the ACE2-binding motif on the opposite side of the domain (Table 2). This progression reflects a refinement of functional influence, transitioning from indirect allostery (F1) to hybrid mechanisms involving both conformational modulation and partial steric hindrance (F2), and finally to direct receptor competition (F3). These shifts are not only structural but also network-determined, highlighting how residue interaction patterns shape neutralization efficacy and escape vulnerability.

### 3.6. Assessing Validity of Computational Predictions

The comprehensive multi-modal computational approach employed in this study generated a wealth of hypotheses regarding the molecular mechanisms of Class 4 antibody neutralization. To ensure the reliability and interpretability of our findings, it was crucial to evaluate the level of confidence associated with different predictions based on their agreement with available experimental data and the internal consistency of our computational methods. This section provides a structured assessment of how our computational results relate to experimental observations and delineates between well-validated conclusions, robust predictions, and novel hypotheses.

Central to establishing confidence in our findings is the strong agreement observed between our computational predictions and independent experimental datasets. This validation is particularly evident in our analysis of key residue interactions and escape mutation profiles. For instance, the critical binding hotspot residues identified through our MM-GBSA energetic decomposition for Class 4 antibodies align precisely with experimentally determined escape mutations from DMS studies. The predicted importance of residues R408, V503, and Y505 for the DH1047 antibody, and D405, R408, and V503/G504 for the S2X259 antibody, in mediating binding and determining escape vulnerability, is directly corroborated by the high-impact mutations observed in DMS experiments [26,27,28,29,30,37,38,39,40,41,42]. Similarly, the mutational scanning analysis effectively captures stability changes associated with key RBD mutations. The predicted mild destabilization effects for mutations like L455S and F456L (ΔΔG ~ 0.5–0.7 kcal/mol) are consistent with experimental observations that group F3 antibodies such as SA55 maintain effectiveness against variants carrying these changes [39,40], underscoring the predictive power of our integrated energetic approach. This convergence between computational predictions and experimental data underscores the quantitative reliability of our integrated energetic approach.

Furthermore, our dynamic network analysis findings are strongly supported by experimental constraints. The identification of a conserved “allosteric ring” within the RBD core, serving as a central communication hub, is validated by its overlap with residues identified as structurally and functionally constrained in DMS studies. The predicted network hubs and communication pathways are often found at positions where mutations are either rare or severely detrimental to RBD function, reinforcing the biological relevance of our network models. The predicted increased flexibility in the RBM loop (~470–490) for Class 4 group F1 antibodies is consistent with experimental kinetic data showing altered ACE2 association and dissociation rates upon CR3022 binding [31,56,57], further bridging our computational dynamics with functional observations.

Beyond direct comparisons with experimental data, the internal consistency of our multi-modal approach provides strong support for several key mechanistic insights. The convergence of evidence from independent coarse-grained and atomistic molecular dynamics simulations, MM-GBSA energetic calculations, and dynamic network modeling consistently points to the same fundamental conclusions regarding the mechanistic continuum among Class 4 antibodies. The predicted shift from core stabilization with RBM mobilization (group F1 antibodies), to intermediate dynamics (group F2 antibodies), and finally to strong RBM locking (group F3 antibodies), is a robust finding supported by multiple orthogonal computational analyses.

However, not all findings reach the same level of validation. Some detailed mechanistic hypotheses, while strongly supported by our computational data, represent novel predictions that await direct experimental confirmation. For example, the precise molecular pathways through which F1 antibodies allosterically enhance RBM flexibility via core stabilization, or the specific role of individual peripheral network connections in dictating the distinct escape profiles of groups F1, F2, and F3 antibodies, constitute testable hypotheses generated by our modeling. The detailed energetic synergy predicted for S2X259 binding, involving specific hydrophobic and electrostatic interactions, also represents a strong computational prediction whose finer details require further experimental scrutiny.

It is also important to acknowledge the inherent limitations of the computational methods used. While our extensive sampling strategy (100 independent 1 μs atomistic trajectories per system) provides robust statistics for the dynamic and energetic properties reported, it may not capture extremely rare conformational states. MM-GBSA, while validated for relative energetic profiles, provides approximate absolute binding free energies and may be subject to systematic errors compared to more computationally demanding methods like MM-PBSA. Our network models offer valuable insights into communication pathways but represent a coarse-grained view of complex dynamic processes. Recognizing these limitations allows for a more nuanced interpretation of the results.

In summary, the strength of our computational study lies in the convergence of evidence from multiple complementary methods and their agreement with independent experimental observations. This framework allows us to clearly distinguish between highly validated results (such as the location of key escape-prone residues and general dynamic trends), well-supported predictions (such as the specific mechanisms of dynamic modulation and network architecture), and novel hypotheses (such as the precise nature of allosteric pathways) that can guide future experimental investigations.

## 4. Discussion

Building upon our detailed computational and validation analyses, the broader implications of presented findings point to a unique molecular strategy employed by Class 4 antibodies. The results detailed molecular mechanisms underlying antibody recognition showing a modular and progressive adaptation among Class 4 antibodies. Despite targeting a shared, conserved cryptic epitope on the RBD, these antibodies have evolved distinct mechanisms that fine-tune the balance between neutralization potency, breadth, and escape resistance. This adaptability is achieved not merely through direct binding but through the strategic modulation of the RBD’s conformational dynamics and allosteric communication networks. The central finding of this study suggests a mechanistic continuum among Class 4 antibodies ranging from indirect allosteric mechanism (group F1 antibodies) to a hybrid mechanism (group F2 antibodies) and a combination of direct receptor blocking with localized allostery (group F3 antibodies).

The functional outcome of antibody binding is fundamentally determined by how the interaction interface propagates signals through the RBD. Group F1 antibodies (CR3022, EY6A, COVA1-16) illustrate a paradigm of indirect neutralization. Their engagement stabilizes the RBD central core while paradoxically increasing the flexibility of the RBM loop. This unique dynamic signature—core rigidity coupled with RBM adaptability—allosterically perturbs the RBD’s functional landscape, favoring conformations that are unfavorable for productive ACE2 engagement. This mechanism, supported by the extensive communication pathways identified through dynamic network analysis, explains their robust cross-reactivity against sarbecoviruses and their resilience to escape, as mutations typically target less critical peripheral residues. As we move along the mechanistic continuum, group F2 antibody DH1047 represents an intriguing intermediate adaptation. Its binding mode extends towards the ACE2 interface, engaging residues such as T376 and R408. This shift is mirrored in its altered dynamic profile, characterized by reduced RBM flexibility. Consequently, DH1047 utilizes a hybrid strategy, merging residual allosteric effects with elements of direct steric hindrance. This dual mechanism enhances its neutralization potency relative to F1 antibodies but concurrently introduces vulnerabilities, exemplified by its sensitivity to escape mutations like T376A and R408S observed in Omicron BA.2. At the endpoint of this continuum, group F3 antibody S2X259 employs a strategy of direct competition. It induces the most pronounced stabilization, effectively locking the RBM loop into a conformation that is incompatible with ACE2 binding. The MM-GBSA energetic analysis confirms that S2X259 binding interactions are underpinned by robust hydrophobic and electrostatic contacts that significantly overlap with the ACE2 footprint. This direct mechanism confers the highest neutralization potency but also the greatest escape risk, as mutations at critical contacting residues (D405, R408) can severely compromise binding.

A unifying principle governing this functional diversification emerges from our dynamic network modeling: a conserved “allosteric ring” within the RBD core serves as a fundamental communication scaffold for all Class 4 antibodies. The distinct functional outcomes arise not from redesigning this core recognition element, but from the antibody-specific refinement of communication pathways that emanate from this ring towards the ACE2 interface. This modular architecture suggests that neutralization strategies evolve through the progressive tuning of these peripheral network connections. This mechanistic understanding illuminates a fundamental trade-off in antibody design: achieving high potency through direct receptor competition inherently increases vulnerability to escape, whereas indirect allostery offers greater durability and breadth but lower potency. These insights have significant implications for therapeutic strategies. Rather than relying on a single antibody, the optimal approach may involve rational combinations that synergistically leverage the stability and breadth of F1 antibodies with the potency of F3 antibodies, potentially using F2 intermediates to bridge these properties for enhanced efficacy and durability against evolving variants.

## 5. Conclusions

This study provides a comprehensive mechanistic framework for understanding how Class 4 antibodies modulate the RBD conformational dynamics and neutralize the virus through indirect or direct interference with ACE2 binding. By integrating structural analysis, MD simulations, binding free energy decomposition, and network-based allosteric modeling, we uncover a progressive evolution in antibody function—from classic allostery (group F1) to hybrid mechanisms involving both dynamic modulation and partial steric hindrance (group F2), and finally to direct competitive inhibition (group F3).

The agreement between DMS experiments, mutational scanning, and MM-GBSA binding free energy calculations underscores the accuracy of computational methods in predicting residue-level contributions to antibody recognition. Importantly, dynamic network analysis reveals a central “allosteric ring” embedded in the RBD core, serving as a conserved communication hub across all Class 4 complexes. Antibody-specific extensions from this core propagate toward the ACE2 interface, forming long-range signaling pathways that influence viral entry without direct physical overlapping. These insights reinforce the idea that neutralization need not rely on direct blocking, but can emerge through conformational redistribution and kinetic modulation, offering a non-canonical yet potent mechanism of action.

The strength of our findings is supported by the excellent agreement between our computational predictions—particularly key binding hotspots and dynamic trends—and independent experimental data, including DMS profiles and structural studies. This convergence validates our approach and distinguishes between highly validated results, well-supported predictions, and novel, testable hypotheses regarding specific allosteric pathways and energetic synergies. While acknowledging the approximations inherent in computational methods, our multi-modal strategy provides a robust and nuanced understanding of antibody function.

Together, the findings of this study support a modular model of antibody-induced allostery where neutralization strategies evolve via refinement of peripheral network connections, rather than complete redesign of the epitope itself. This evolutionary logic aligns with fundamental principles of protein allostery, reinforcing that functionally distant sites can exert meaningful influence through network-level interactions. Collectively, these insights illuminate a fundamental trade-off in antibody design: achieving high potency through direct receptor competition inherently increases escape vulnerability, whereas indirect allostery offers greater durability and breadth but lower potency. These findings may have implications for therapeutic development, suggesting that optimal strategies may involve rational combinations of antibodies leveraging different mechanisms (e.g., group F1 antibodies for breadth/stability, group F3 antibodies for potency) to achieve broad, potent, and durable protection against evolving sarbecoviruses. Furthermore, the identification of conserved dynamic networks and allosteric mechanisms offers potential avenues for designing next-generation therapeutics that target these critical communication pathways, enhancing the potential to combat immune escape.

## Figures and Tables

**Table 1:** Summary of the structural and dynamics analysis for Class 4 antibodies.

	Group F1 Antibodies	Group F2 Antibodies	Group F3 Antibodies
**Binding Site**	Core RBD, cryptic	Partial overlap + ACE2 interface	Further shift toward ACE2 interface
**ACE2 Competition**	No	Partial	Yes
**Key Interactions**	Leu445, Phe486, Tyr505	R408, 500–508	D405, R408, V503, G504, Y508
**Escape Mutations**	383–386, 390, 391	408, 500–508	501, 505
**RMSF Profile**	Stabilized core, flexible 470–490 loop	Moderate flexibility in 450–470	Reduced flexibility in 470–490
**Neutralization Mechanism**	Allosteric	Partial steric hindrance	Direct competition with ACE2

**Table 2:** Comparative network architecture for group F1, F2, and F3 antibodies ranging from broad modulation to targeted interference.

Feature	Group F1	Group F2	Group F3
**Network Localization**	Broad, diffuse	Intermediate, partially localized	Highly localized
**ACE2 Coupling**	Weak, indirect	Moderate, hybrid	Strong, direct
**Key Residues**	Core β-sheet (e.g., 355–380, 431, 436)	Core + emerging ACE2 sites (T376, R408, V503)	ACE2-overlapping residues (D405, R408, G504, Y508)
**Escape Vulnerability**	Low	Moderate	Moderate
**Neutralization Mechanism**	Indirect allostery	Hybrid (dynamic + partial steric)	Direct ACE2 competition + allosteric stabilization

## Data Availability

Data is fully contained within the article and Supplementary Materials. Crystal structures were obtained and downloaded from the Protein Data Bank (http://www.rcsb.org, accessed on 20 February 2025). The rendering of protein structures was carried out with the UCSF ChimeraX package (https://www.rbvi.ucsf.edu/chimerax/, accessed on 15 February 2025) and Pymol (https://pymol.org/2/, accessed on 11 February 2025). All mutational heatmaps were produced using the developed software that is freely available at https://alshahrani.shinyapps.io/HeatMapViewerApp/ (accessed on 16 March 2025).

## Supplementary Materials

The following supporting information can be downloaded at: https://www.mdpi.com/article/10.3390/biom15091340/s1, Figure S1: Overlap of Binding Hotspots Among Class 4 RBD Antibodies Across Groups F1 and F2; Figure S2: Structural Epitope Mapping of Group F1 Class 4 Antibodies Binding to the RBD; Figure S3: Binding Free Energy Decomposition of Group F1 Antibodies with the RBD Using MM-GBSA Analysis; Figure S4: Binding Free Energy Decomposition of Group F2 and F3 Antibodies with the RBD Using MM-GBSA Analysis; Table S1: The list of the intermolecular contacts in the structure of the CR3022 complex with RBD (pdb id 6YM0); Table S2: The list of the intermolecular contacts in the structure of the EY6A complex with RBD (pdb id 7ZF3); Table S3: The list of the intermolecular contacts in the structure of the COVA1-16 complex with RBD (pdb id 7JMW); Table S4: The list of the intermolecular contacts in the structure of the DH1047 complex with RBD (pdb id 8DTK); Table S5: The list of the intermolecular contacts in the structure of the S2X259 complex with RBD (pdb id 7RAL).
