# Supplementary material for: Allosteric Control Overcomes Steric Limitations for Neutralizing Antibodies Targeting Conserved Binding Epitopes of the SARS-CoV-2 Spike Protein: Exploring the Intersection of Binding, Allostery, and Immune Escape with a Multimodal Computational Approach
Source: Biomolecules. 2025 Sep 18;15(9):1340. doi: 10.3390/biom15091340 (PMC12466954; doi:10.3390/biom15091340)
Supplement: Supplementary file 1 [file biomolecules-15-01340-s001.zip › biomolecules-3781359-supplementary.pdf]

Supplementary Materials

Allosteric Control Overcomes Steric Limitations for Neutralizing Antibodies Targeting Conserved Binding Epitopes of the SARS-CoV-2 Spike Protein: Exploring the Intersection of Binding, Allostery, and Immune Escape with a Multimodal Computational Approach

Mohammed Alshahrani [1], Vedant Parikh [1], Brandon Foley [1] and Gennady Verkhivker [1,2,*]

[1] Keck Center for Science and Engineering, Graduate Program in Computational and Data Sciences, Schmid College of Science and Technology, Chapman University, Orange, CA 92866, USA; alshahrani@chapman.edu (M.A.); vedpar31@gmail.com (V.P.); brfoley@chapman.edu (B.F.)
[2] Department of Biomedical and Pharmaceutical Sciences, Chapman University School of Pharmacy, Irvine, CA 92618, USA
* Correspondence: verkhivk@chapman.edu; Tel.: +1-714-516-4586

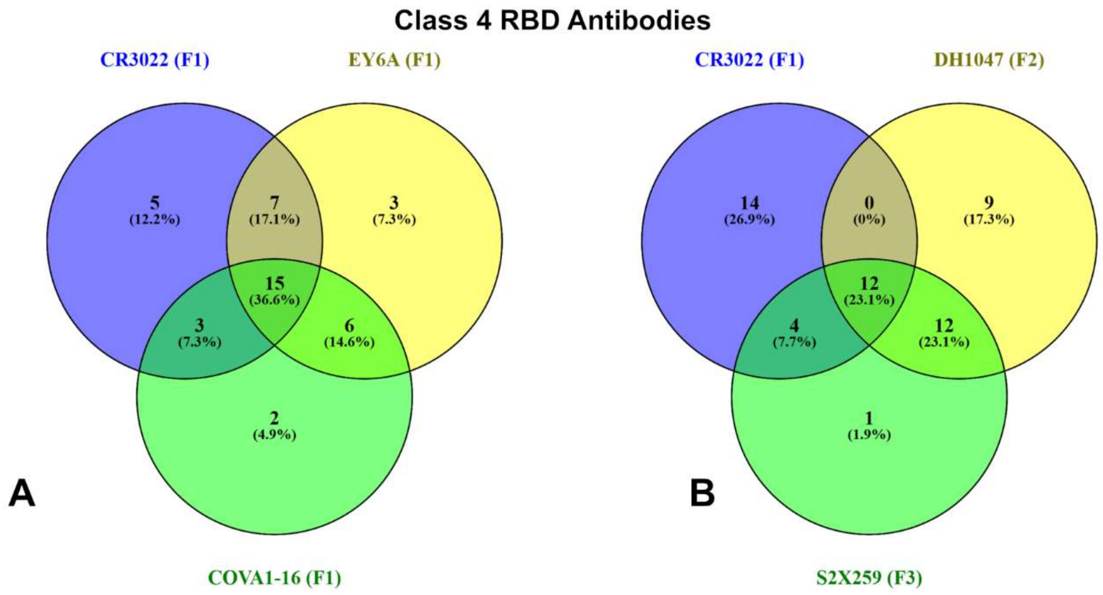

Figure S1. Overlap of Binding Hotspots Among Class 4 RBD Antibodies Across Groups F1 and F2. (A) Venn diagram illustrates the overlap in binding hotspots among group F1 class 4 antibodies CR3022, EY6A, and COVA1-16. The diagram shows the number of residues (and their percentage contributions) that are shared or unique to each antibody within this group. For example, CR3022 (F1) shares 15 residues (36.6%) with both EY6A and COVA1-16, while 5 residues (12.2%) are unique to CR3022. (B) Venn diagram comparing the binding hotspots between group F1 antibody CR3022 (F1) and group F2 antibody DH1047 (F2). This comparison highlights the transition from indirect allostery to partial steric hindrance as observed in group F2. Notably, DH1047 (F2) exhibits a larger set of unique binding hotspots (14 residues, 26.9%) compared to its overlap with CR3022 (4 residues, 7.7%), reflecting its more direct engagement with the ACE2 interface. The green circle represents the overlapping residues common to all three groups, indicating conserved interactions across these antibodies. The percentages indicate the relative contribution of each subset of residues to the total binding hotspots identified for each antibody.

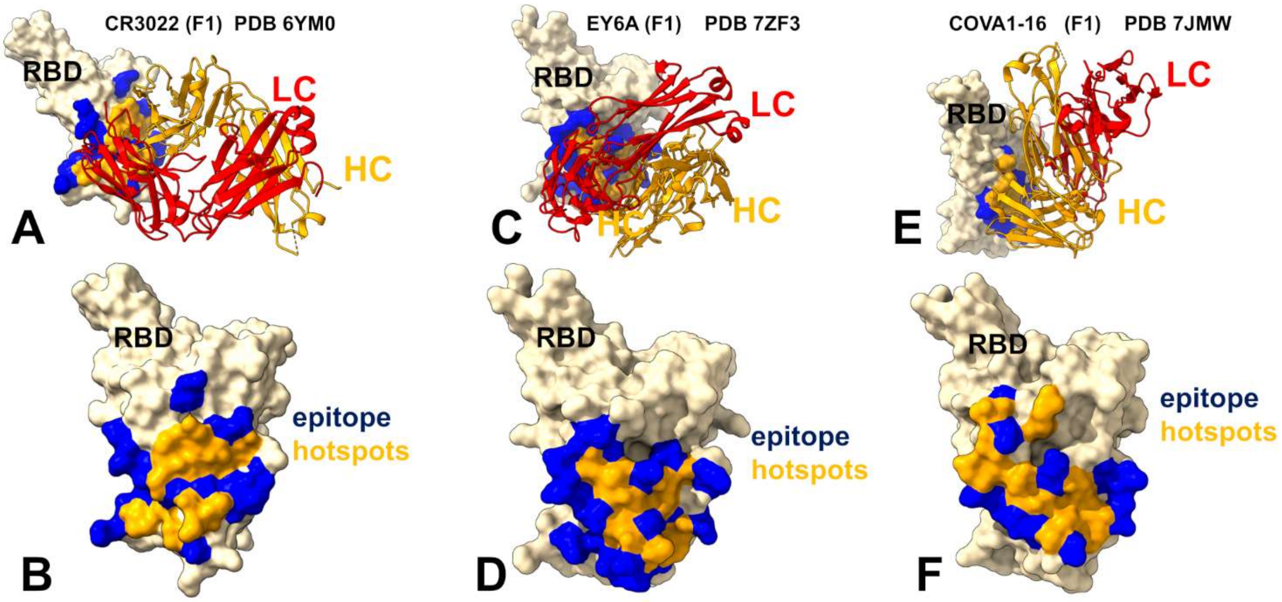

Figure S2. Structural Epitope Mapping of Group F1 Class 4 Antibodies Binding to the RBD. The structures and epitope hotspots for three representative group F1 class 4 antibodies — CR3022 (PDB ID: 6YM0), EY6A (PDB ID: 7ZF3), and COVA1-16 (PDB ID: 7JMW). The three-dimensional structures of the RBD–CR3022 complex (A), RBD-EY6A complex (C) and COVA1-16/RBD (F). The RBD is depicted in wheat-colored surface, with the heavy chain (HC) of the antibody in orange and the light chain (LC) in red. The binding epitope residues are shown in blue surface and the RBD binding hotspots are shown in orange surface. Panels B, D, and F provide a detailed view of the RBD, the epitope and binding hotspots for CR3022 (PDB ID: 6YM0), EY6A (PDB ID: 7ZF3), and COVA1-16 (PDB ID: 7JMW) respectively. The epitope sites are highlighted in blue surface, and binding hotspots are in orange surface. These panels reveal that while all three antibodies target a deeply conserved hydrophobic core within the RBD, there are subtle differences in their contact footprints and residue-specific interactions, reflecting minor variations in orientation and binding geometry.

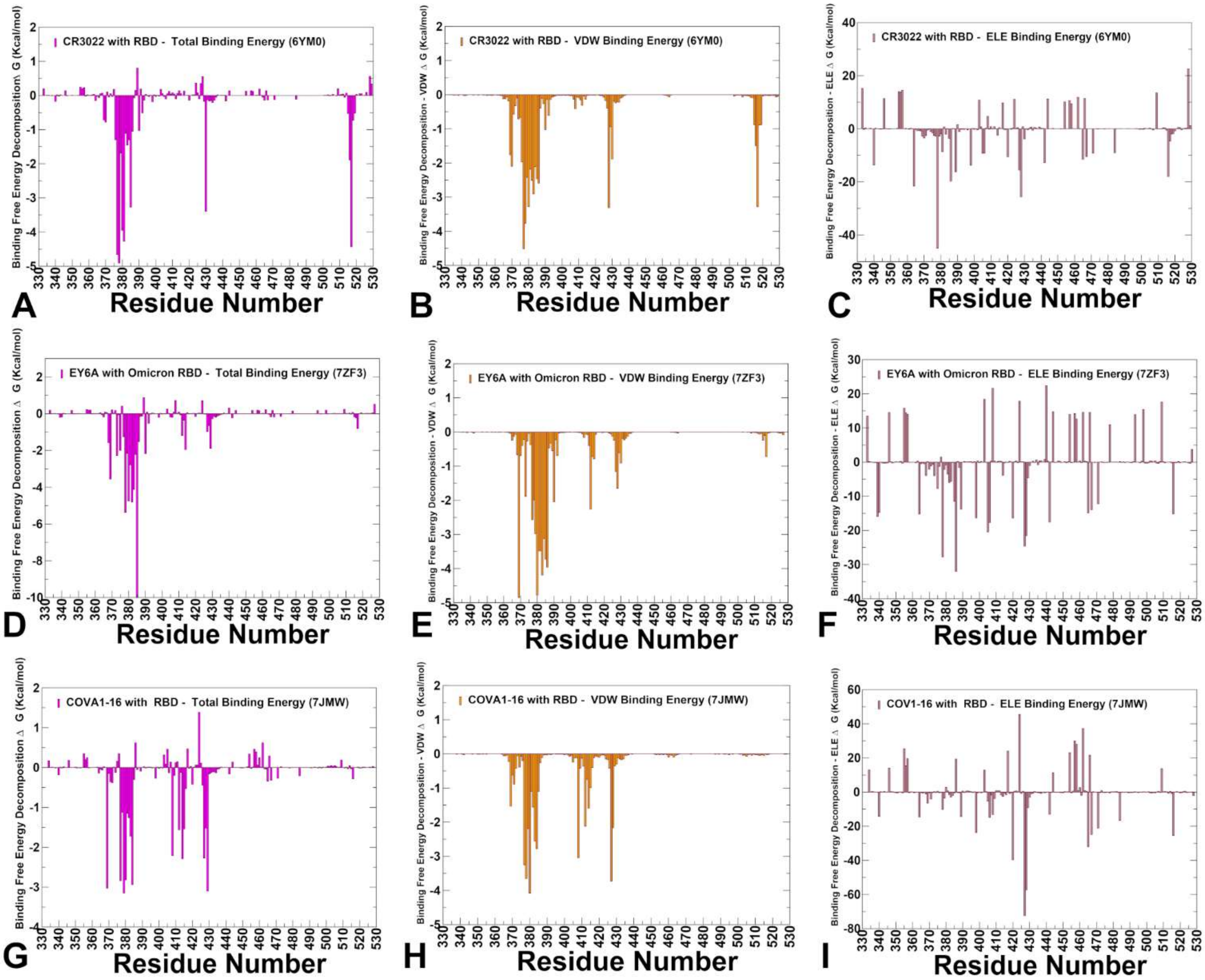

Figure S3. Binding Free Energy Decomposition of Group F1 Antibodies with the RBD Using MM-GBSA Analysis. MM-GBSA binding free energy decomposition for three representative group F1 class 4 antibodies — CR3022, EY6A, and COVA1-16 in complex with RBD. (A) The total binding free energy for CR3022 binding to the RBD, highlighting key residues contributing to overall stability. (B) Van der Waals (VDW) component of the binding free energy, showing dominant contributions from hydrophobic interactions. (C) Electrostatic (ELE) component of the binding free energy, revealing residues critical for charge-based stabilization. (D) The total binding free energy for EY6A binding to the RBD, emphasizing energetic contributions across the interface. (E) VDW contribution to binding, illustrating the role of nonpolar contacts in stabilizing the complex. (F) ELE contribution to binding, identifying residues involved in electrostatic interactions. (G) The total binding free energy for COVA1-16 binding to the RBD. (H) VDW component of the binding free energy. (I) ELE component of the binding free energy, pinpointing residues crucial for electrostatic complementarity. Residue-based binding free energy values are shown for total energies as magenta-colored filled bars, van der Waals contributions as orange-colored bars and electrostatic contributions as light-brown colored bars. MM-GBSA contributions are evaluated using 1,000 samples from MD simulations. The standard error of the mean (SEM) calculated from the ensemble of snapshots used in the MM-GBSA analysis (n=1,000 samples). The SEM values for the binding free energy range from 0.12 to 0.18 kcal/mol for the interfacial RBD residues and 0.08 to 0.15 kcal/mol for the interfacial heavy chain residues.

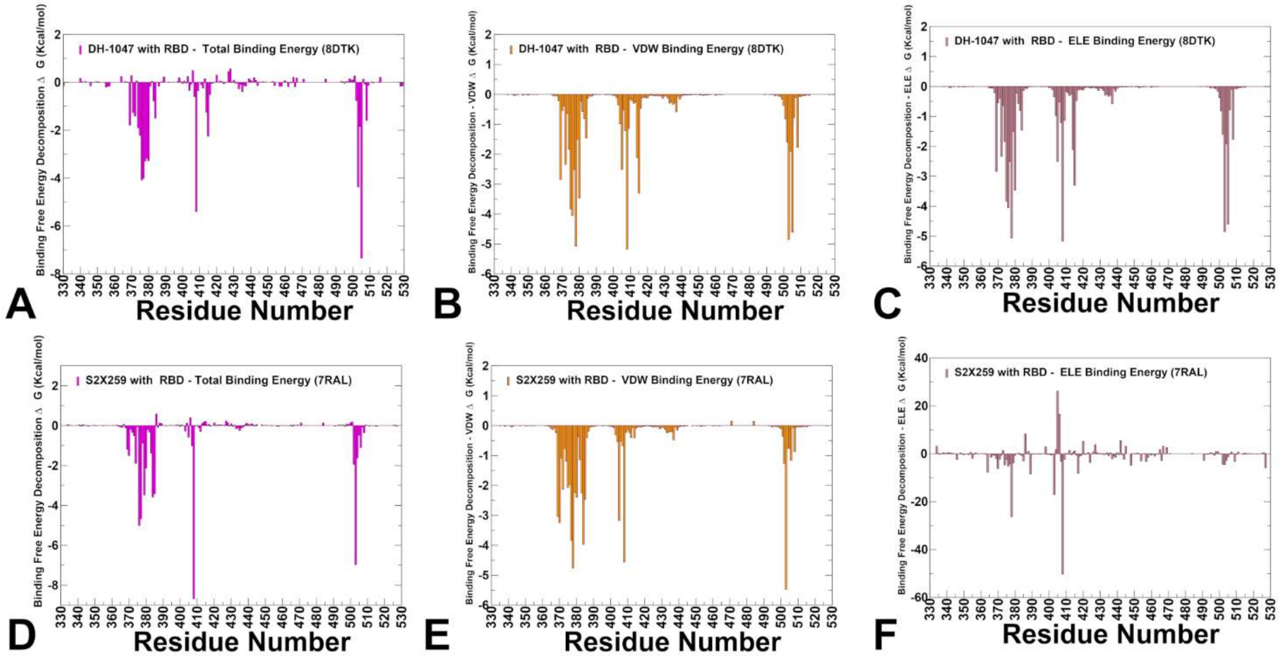

Figure S4. Binding Free Energy Decomposition of Group F2 and F3 Antibodies with the RBD Using MM-GBSA Analysis. MM-GBSA binding free energy decomposition for group F2 DH1047 and group F3 S2X259 in complex with RBD. (A) The total binding free energy for DH1047 binding to the RBD, highlighting key residues contributing to overall stability. (B) Van der Waals (VDW) component of the binding free energy, showing dominant contributions from hydrophobic interactions. (C) Electrostatic (ELE) component of the binding free energy, revealing residues critical for charge-based stabilization. (D) The total binding free energy for S2X259 binding to the RBD (E) VDW contribution to binding, illustrating the role of nonpolar contacts in stabilizing the complex. (F) ELE contribution to binding, identifying residues involved in electrostatic interactions. Residue-based binding free energy values are shown for total energies as magenta-colored filled bars, van der Waals contributions as orange-colored bars and electrostatic contributions as light-brown colored bars. MM-GBSA contributions are evaluated using 1,000 samples from MD simulations. The standard error of the mean (SEM) are calculated from the ensemble of snapshots used in the MM-GBSA analysis (n=1,000 samples). The SEM values for the binding free energy range from 0.08 to 0.15 kcal/mol for the interfacial RBD residues and 0.11 to 0.17 kcal/mol for the interfacial heavy chain residues.

Table S1. The list of the intermolecular contacts in the structure of the CR3022 complex with RBD (pdb id 6YM0).

RBD Residue	RBD Residue Number	RBD chain	Ab Residue	Ab Residue Number	Ab chain
TYR	369	E	ILE	30	H
TYR	369	E	THR	31	H
TYR	369	E	GLY	28	H
TYR	369	E	PHE	29	H
TYR	369	E	TYR	27	H
ASN	370	E	GLY	28	H
ASN	370	E	TYR	27	H
SER	371	E	ILE	30	H
PHE	374	E	ILE	30	H
SER	375	E	TYR	52	H
SER	375	E	ILE	30	H
SER	375	E	GLY	54	H
THR	376	E	ILE	30	H
THR	376	E	TYR	52	H
THR	376	E	GLY	54	H
THR	376	E	ASP	55	H
PHE	377	E	THR	31	H
PHE	377	E	TRP	33	H
PHE	377	E	TYR	52	H
PHE	377	E	TYR	32	H
PHE	377	E	ILE	30	H
LYS	378	E	ILE	30	H
LYS	378	E	THR	31	H
LYS	378	E	TRP	33	H
LYS	378	E	ASP	55	H
LYS	378	E	GLU	57	H
LYS	378	E	TYR	52	H
CYS	379	E	THR	31	H
CYS	379	E	TRP	33	H
CYS	379	E	ILE	102	H
CYS	379	E	SER	100	H
CYS	379	E	GLY	101	H
TYR	380	E	TRP	33	H
TYR	380	E	ARG	59	H

TYR	380	E	THR	104	H
TYR	380	E	ILE	102	H
TYR	380	E	GLU	57	H
TYR	380	E	GLY	101	H
TYR	380	E	SER	103	H
GLY	381	E	ILE	34	L
GLY	381	E	TYR	31	L
GLY	381	E	THR	104	H
GLY	381	E	TYR	38	L
GLY	381	E	GLY	101	H
GLY	381	E	ILE	102	H
GLY	381	E	SER	103	H
GLY	381	E	TRP	56	L
VAL	382	E	GLY	101	H
VAL	382	E	ILE	102	H
VAL	382	E	SER	103	H
VAL	382	E	SER	100	H
VAL	382	E	TRP	56	L
VAL	382	E	ILE	34	L
VAL	382	E	THR	104	H
VAL	382	E	TYR	38	L
SER	383	E	GLY	101	H
SER	383	E	SER	100	H
SER	383	E	GLY	99	H
SER	383	E	THR	104	H
SER	383	E	PRO	105	H
PRO	384	E	SER	100	H
PRO	384	E	THR	31	H
PRO	384	E	GLY	101	H
THR	385	E	TYR	32	H
THR	385	E	THR	31	H
THR	385	E	SER	100	H
THR	385	E	GLN	1	H
THR	385	E	ASP	107	H
LYS	386	E	PRO	105	H
LYS	386	E	TYR	55	L
LYS	386	E	GLU	61	L
LYS	386	E	LEU	52	L
LYS	386	E	ASP	107	H
LYS	386	E	SER	100	H
ASP	389	E	TYR	55	L
LEU	390	E	TRP	56	L
PHE	392	E	TRP	56	L

PHE	392	E	ILE	34	L
ARG	408	E	ASP	55	H
ASP	427	E	TYR	31	L
ASP	428	E	SER	32	L
ASP	428	E	TYR	31	L
ASP	428	E	SER	33	L
ASP	428	E	TYR	98	L
PHE	429	E	TYR	31	L
THR	430	E	ILE	34	L
THR	430	E	TYR	31	L
THR	430	E	SER	33	L
THR	430	E	TYR	38	L
PHE	515	E	SER	33	L
PHE	515	E	ILE	34	L
GLU	516	E	ILE	34	L
GLU	516	E	SER	33	L
LEU	517	E	ILE	34	L
LEU	517	E	ASN	35	L
LEU	517	E	SER	32	L
LEU	517	E	SER	33	L
LEU	517	E	LYS	36	L
LEU	518	E	SER	33	L
HIS	519	E	ASN	35	L

Table S2. The list of the intermolecular contacts in the structure of the EY6A complex with RBD (pdb id 7ZF3).

RBD Residue	RBD Residue Number	RBD chain	Ab Residue	Ab Residue Number	Ab chain
LEU	368	E	TYR	59	H
LEU	368	E	ASN	57	H
TYR	369	E	ASN	57	H
TYR	369	E	LYS	58	H
TYR	369	E	SER	56	H
TYR	369	E	TYR	59	H
ASN	370	E	LYS	58	H
ASN	370	E	SER	56	H
ASN	370	E	TYR	59	H
ASN	370	E	ASN	57	H
ALA	372	E	LYS	65	H
PRO	373	E	GLY	66	H
PRO	373	E	LYS	65	H
PHE	375	E	LYS	65	H
THR	376	E	LYS	65	H
PHE	377	E	TYR	59	H
PHE	377	E	LYS	65	H
PHE	377	E	LEU	95	L
LYS	378	E	LEU	95	L
LYS	378	E	SER	93	L
LYS	378	E	ALA	96	L
LYS	378	E	ASP	1	L
LYS	378	E	ASP	62	H
CYS	379	E	LEU	95	L
CYS	379	E	SER	93	L
CYS	379	E	TYR	92	L
CYS	379	E	THR	94	L
TYR	380	E	SER	93	L
TYR	380	E	TYR	92	L
TYR	380	E	THR	94	L
GLY	381	E	SER	93	L
GLY	381	E	TYR	92	L

GLY	381	E	THR	94	L
GLY	381	E	TRP	104	H
GLY	381	E	SER	91	L
GLY	381	E	TYR	32	L
GLY	381	E	VAL	105	H
VAL	382	E	TRP	104	H
VAL	382	E	TYR	32	L
VAL	382	E	VAL	105	H
VAL	382	E	TYR	92	L
VAL	382	E	THR	94	L
SER	383	E	TRP	104	H
SER	383	E	VAL	105	H
SER	383	E	THR	94	L
SER	383	E	TYR	106	H
PRO	384	E	TYR	59	H
PRO	384	E	THR	94	L
PRO	384	E	TYR	106	H
PRO	384	E	LEU	95	L
PRO	384	E	ASN	57	H
THR	385	E	VAL	50	H
THR	385	E	TYR	53	H
THR	385	E	TYR	106	H
THR	385	E	SER	52	H
THR	385	E	TYR	59	H
THR	385	E	ASP	33	H
THR	385	E	ASN	57	H
THR	385	E	ILE	51	H
LYS	386	E	TRP	104	H
LYS	386	E	ASP	33	H
LYS	386	E	GLY	101	H
LYS	386	E	VAL	105	H
LYS	386	E	LEU	103	H
LYS	386	E	ASP	99	H
LYS	386	E	TYR	106	H
LYS	386	E	LYS	102	H
ASN	388	E	TYR	53	H
ASP	389	E	TYR	53	H
LEU	390	E	TRP	104	H
PHE	392	E	TRP	104	H
ALA	411	E	GLN	27	L
PRO	412	E	GLN	27	L
PRO	412	E	TYR	92	L
GLY	413	E	GLN	27	L

GLN	414	E	GLN	27	L
PRO	426	E	TYR	92	L
ASP	427	E	SER	28	L
ASP	427	E	SER	30	L
ASP	427	E	TYR	92	L
ASP	428	E	SER	30	L
ASP	428	E	TYR	92	L
PHE	429	E	TYR	92	L
THR	430	E	TYR	92	L
THR	430	E	TRP	104	H
LEU	517	E	TRP	104	H

Table S3. The list of the intermolecular contacts in the structure of the COVA1-16 complex with RBD (pdb id 7JMW).

RBD Residue	RBD Residue Number	RBD chain	Ab Residue	Ab Residue Number	Ab chain
LEU	368	A	ARG	100	H
TYR	369	A	ARG	100	H
ASN	370	A	ARG	100	H
SER	371	A	ARG	100	H
ALA	372	A	ARG	100	H
PHE	374	A	ARG	100	H
PHE	377	A	TYR	100	H
PHE	377	A	TYR	99	H
PHE	377	A	ARG	100	H
LYS	378	A	TYR	100	H
LYS	378	A	TYR	99	H
CYS	379	A	ASN	98	H
CYS	379	A	TYR	100	H
CYS	379	A	TYR	99	H
TYR	380	A	ARG	97	H
TYR	380	A	TYR	100	H
TYR	380	A	TYR	99	H
TYR	380	A	ASN	98	H
GLY	381	A	ASN	98	H
GLY	381	A	ARG	97	H
GLY	381	A	TYR	100	H
VAL	382	A	TYR	100	H
SER	383	A	TYR	100	H
SER	383	A	GLY	100	H
PRO	384	A	TYR	100	H
PRO	384	A	ARG	100	H
PRO	384	A	GLY	100	H
THR	385	A	ARG	100	H
THR	385	A	GLY	100	H
ARG	408	A	LEU	54	L
ARG	408	A	ASN	53	L
ARG	408	A	TYR	49	L
PRO	412	A	ARG	97	H
PRO	412	A	PRO	96	H

PRO	412	A	TYR	32	H
GLY	413	A	GLN	101	H
GLY	413	A	TYR	32	H
GLY	413	A	ARG	94	H
GLY	413	A	HIS	102	H
GLN	414	A	PRO	96	H
GLN	414	A	GLN	101	H
GLN	414	A	GLU	55	L
GLN	414	A	TYR	32	H
GLN	414	A	TYR	49	L
THR	415	A	THR	56	L
GLY	416	A	THR	56	L
PRO	426	A	ARG	97	H
ASP	427	A	TYR	32	H
ASP	427	A	THR	28	H
ASP	427	A	GLY	26	H
ASP	427	A	ARG	97	H
ASP	427	A	SER	31	H
ASP	427	A	TYR	27	H
ASP	428	A	THR	28	H
ASP	428	A	ARG	97	H
ASP	428	A	SER	31	H
PHE	429	A	ARG	97	H
THR	430	A	ARG	97	H

Table S4. The list of the intermolecular contacts in the structure of the DH1047 complex with RBD (pdb id 8DTK).

RBD Residue	RBD Residue Number	RBD chain	Ab Residue	Ab Residue Number	Ab chain
TYR	369	A	GLY	100	C
TYR	369	A	TYR	52	C
TYR	369	A	ASN	56	C
ASN	370	A	GLY	54	C
SER	371	A	ASN	56	C
ALA	372	A	ASN	56	C
ALA	372	A	THR	57	C
ALA	372	A	GLY	55	C
ALA	372	A	GLY	54	C
SER	373	A	ASN	56	C
PHE	374	A	ASN	56	C
SER	375	A	ASN	56	C
SER	375	A	LEU	100	C
SER	375	A	THR	57	C
SER	375	A	ASP	100	C
SER	375	A	ILE	58	C
THR	376	A	LEU	100	C
THR	376	A	ASP	100	C
PHE	377	A	ASP	100	C
PHE	377	A	GLY	100	C
LYS	378	A	TRP	100	C
LYS	378	A	GLY	100	C
LYS	378	A	ASP	100	C
CYS	379	A	GLY	100	C
CYS	379	A	TRP	100	C
TYR	380	A	TRP	100	C
PRO	384	A	GLY	100	C
GLY	404	A	LEU	100	C
ASP	405	A	GLN	27	B
ASP	405	A	SER	93	B
ASP	405	A	TYR	92	B
VAL	407	A	LEU	100	C

ARG	408	A	LEU	100	C
ARG	408	A	TYR	27	B
ARG	408	A	SER	93	B
ARG	408	A	TYR	32	B
ARG	408	A	TYR	92	B
ARG	408	A	TYR	91	B
GLN	409	A	TYR	27	B
GLN	409	A	TYR	92	B
GLY	413	A	SER	27	B
GLN	414	A	TRP	100	C
GLN	414	A	TYR	27	B
GLN	414	A	ASN	28	B
GLN	414	A	SER	27	B
THR	415	A	TYR	27	B
THR	415	A	SER	27	B
GLY	416	A	SER	27	B
ALA	435	A	LEU	100	C
ASN	437	A	ILE	58	C
PRO	499	A	GLN	61	C
THR	500	A	GLN	61	C
ASN	501	A	ASP	1	B
ASN	501	A	GLN	61	C
GLY	502	A	PRO	95	B
GLY	502	A	ASP	1	B
GLY	502	A	GLN	61	C
VAL	503	A	LEU	94	B
VAL	503	A	ILE	58	C
VAL	503	A	TYR	59	C
VAL	503	A	TRP	47	C
VAL	503	A	ASP	1	B
VAL	503	A	GLN	61	C
VAL	503	A	LEU	100	C
VAL	503	A	ALA	60	C
VAL	503	A	PRO	95	B
GLY	504	A	LEU	94	B
GLY	504	A	PRO	95	B
GLY	504	A	SER	93	B
GLY	504	A	LEU	100	C
TYR	505	A	ILE	2	B
TYR	505	A	LEU	94	B
TYR	505	A	GLN	27	B
TYR	505	A	ASP	1	B
TYR	505	A	SER	93	B

GLN	506	A	GLN	61	C
GLN	506	A	GLN	64	C
TYR	508	A	LEU	100	C
TYR	508	A	ILE	58	C
TYR	508	A	LEU	94	B

Table S5. The list of the intermolecular contacts in the structure of the S2X259 complex with RBD (pdb id 7RAL).

RBD Residue	RBD Residue Number	RBD chain	Ab Residue	Ab Residue Number	Ab chain
TYR	369	B	ILE	52	H
TYR	369	B	SER	55	H
TYR	369	B	TRP	107	H
TYR	369	B	MET	54	H
ASN	370	B	LYS	74	H
ASN	370	B	MET	54	H
ASN	370	B	SER	55	H
SER	371	B	SER	55	H
ALA	372	B	SER	55	H
ALA	372	B	MET	57	H
SER	373	B	MET	57	H
PHE	374	B	TRP	107	H
PHE	374	B	SER	55	H
PHE	374	B	MET	57	H
PHE	374	B	ILE	52	H
SER	375	B	ILE	52	H
SER	375	B	GLY	108	H
SER	375	B	ARG	50	H
SER	375	B	ASP	109	H
SER	375	B	TRP	107	H
THR	376	B	TRP	107	H
THR	376	B	ASP	109	H
THR	376	B	GLY	108	H
PHE	377	B	TYR	105	H
PHE	377	B	TRP	107	H
PHE	377	B	GLY	108	H
PHE	377	B	GLY	106	H
PHE	377	B	TYR	104	H
LYS	378	B	TYR	104	H
LYS	378	B	TYR	105	H
LYS	378	B	TRP	107	H
LYS	378	B	GLY	108	H
LYS	378	B	GLY	106	H
CYS	379	B	GLY	106	H
CYS	379	B	TYR	104	H
CYS	379	B	TYR	105	H
CYS	379	B	ASN	103	H

TYR	380	B	TYR	104	H
TYR	380	B	TYR	105	H
TYR	380	B	ASN	103	H
GLY	381	B	TYR	105	H
VAL	382	B	TYR	105	H
SER	383	B	TYR	105	H
SER	383	B	TYR	32	H
SER	383	B	PHE	29	H
PRO	384	B	TYR	105	H
PRO	384	B	TRP	107	H
PRO	384	B	TYR	32	H
PRO	384	B	MET	54	H
PRO	384	B	GLY	106	H
THR	385	B	MET	54	H
THR	385	B	TYR	32	H
THR	385	B	PHE	29	H
GLY	404	B	ASP	109	H
GLY	404	B	TYR	33	L
ASP	405	B	ASN	26	L
ASP	405	B	ALA	31	L
ASP	405	B	SER	95	L
ASP	405	B	TYR	33	L
GLU	406	B	TYR	33	L
VAL	407	B	ASP	109	H
VAL	407	B	TYR	33	L
ARG	408	B	ASP	34	L
ARG	408	B	TYR	33	L
ARG	408	B	ASP	110	H
ARG	408	B	GLY	32	L
ARG	408	B	ALA	31	L
ASN	501	B	LEU	97	L
ASN	501	B	SER	98	L
GLY	502	B	SER	98	L
GLY	502	B	GLY	99	L
GLY	502	B	SER	95	L
GLY	502	B	SER	96	L
GLY	502	B	LEU	97	L
VAL	503	B	PRO	100	L
VAL	503	B	SER	98	L
VAL	503	B	GLY	99	L
VAL	503	B	SER	96	L
VAL	503	B	LEU	97	L
VAL	503	B	SER	95	L

VAL	503	B	TYR	93	L
VAL	503	B	ASP	94	L
GLY	504	B	SER	95	L
GLY	504	B	SER	96	L
TYR	505	B	SER	95	L
GLN	506	B	GLY	99	L
GLN	506	B	SER	98	L
TYR	508	B	TYR	93	L
TYR	508	B	PRO	100	L
